# Effect of Gaseous Ozone and Storage Time on Polyphenolic Profile and Sugar Content in Fruits of Selected *Vaccinium corymbosum* L. Genotypes

**DOI:** 10.3390/molecules28248106

**Published:** 2023-12-15

**Authors:** Józef Gorzelany, Ireneusz Kapusta, Stanisław Pluta, Justyna Belcar, Katarzyna Pentoś, Oskar Basara

**Affiliations:** 1 Department of Food and Agriculture Production Engineering, University of Rzeszów, St. Zelwerowicza 4, 35-601 Rzeszow, Poland; jgorzelany@ur.edu.pl (J.G.); justyna.belcar@op.pl (J.B.); oskarb@dokt.ur.edu.pl (O.B.); 2 Department of Food Technology and Human Nutrition, University of Rzeszów, St. Zelwerowicza 4, 35-601 Rzeszow, Poland; 3 Department of Horticultural Crop Breeding, The National Institute of Horticultural Research, Konstytucji 3 Maja 1/3 Street, 96-100 Skierniewice, Poland; stanislaw.pluta@inhort.pl; 4 Institute of Agricultural Engineering, Wrocław University of Environmental and Life Sciences, 37b Chełmonskiego Street, 51-630 Wroclaw, Poland

**Keywords:** highbush blueberry, ozone fumigation, polyphenol profile, sugar content, storage time

## Abstract

One of the best sources of antioxidant and health-promoting bioactive substances is the fruit of *V. corymbosum*. A potent oxidizing agent, ozone (O_3_), can effectively eliminate bacteria. The application of ozone gas to *V. corymbosum* fruit during storage had a favorable impact on the fruit’s phenolic component and sugar content in the current investigation. After 7 days of storage, phenolic content in all highbush blueberry cultivars and clones tested increased on average by 28.60%, including anthocyanins by 34%. After 14 days of storage, an average increase of 16.50% in phenolic compounds was observed, including a 20.53% increase in anthocyanins. Among all the tested varieties, clone BOR-21 treated with a dose of 0.01 mL·L^−1^ ozone for 30 min after 14 days had the highest TPC—143.73 mg·100 g^−1^ f.w. The sugar content of berries treated with a dose of 0.01 mL·L^−1^ ozone for 30 min, on day 7 and day 14 of storage increased by 9.2% and 6.3%, respectively. On day 7, the highest amount of total sugar (22.74 g·100 g^−1^) was observed in Duke cultivar after being exposed to 0.01 mL·L^−1^ ozone for 15 min. The ozonation treatments enhanced the fruit’s saturation with nutrients, which raises the fruit’s value as food.

## 1. Introduction

The highbush blueberry belongs to the genus *Vaccinium* L., which includes about 150–450 species native to the eastern part of North America. In the early 20th century, breeding and selection of highbush blueberry species was carried out in the USA. The main species in commercial cultivation is the tetraploid (2*n* = 4x = 48)—*Vaccinium corymbosum* L. The highbush blueberry is a shrub that grows up to 3 m tall, has a shallow root system with branches, and upright stems that emerge from buds that form at the shrub’s base. Depending on the spring weather, flowering in Poland often starts in the first or second week of May and lasts for 3 to 4 weeks. The fruit is spherical or egg-shaped, blue-black in color, and has a distinctive bloom. It weighs between 1.0 and 2.5 g and measures 10 to 20 mm in diameter [1,2,3].

Highbush blueberries (*V. corymbosum*) are regarded as one of the most beneficial sources of bioactive substances with antioxidant and health-improving properties. The development of cancer-causing cells can be slowed, inflammation can be reduced, and obesity can be avoided. They can also inhibit and prevent heart disease and diabetes [4,5]. Climate, location of cultivation, amount of sunlight, soil quality, water availability, as well as fruit variety and maturity all play a significant role in determining the phenolic profile [6]. The total amount of phenolic compounds in the fruit is 170.7–320.9 mg GAE·100 g^−1^ f.w. [7]. The anthocyanins that give the fruit its reddish-blue color, including Cyanidin-3-O-glucoside (C3G), Malvidin-3-O-glucoside (M3Gl), and Malvidin-3-O-galactoside (M3Ga), are abundant in the fruit. The flavanol group of substances, including quercetin 3-O-glucoside and chlorogenic acid are both found in the fruit of *V. corymbosum* [2,8,9]. Fruits of the highbush blueberry plant may have 1.2–1.9 g·100 g^−1^ of glucose, 1.0–1.8 g·100 g^−1^ of fructose, and 0.32–0.97 g·100 g^−1^ of sucrose make up their composition [10].

Highbush blueberries are employed as an exceptional dessert fruit due to the rise in production, the enthusiasm of growers and customers, and the abundance of nutrients and health-improving components. They are also a highly valuable raw material in the culinary sector used to make tinctures, jams, juices, nectars, frozen meals, and other products that, depending on the processing technique, have strong antioxidant activity. According to published studies, the amount of health-promoting substances, primarily anthocyanins, appears to decrease with longer storage times. Compared to jams stored at 22 °C, jams stored at 6 °C for 8 months had a four- to sevenfold higher anthocyanin content. The high-sugar products also showed a slower decline in bioactive chemicals [2].

Ozone (O_3_), an agent with strong oxidizing capabilities, is most frequently utilized in gaseous form. Without affecting the fruit’s quality, ozonation is an environmentally friendly, nonthermal method of food preservation [11,12]. By dissolving the necessary for function phospholipid molecules and proteins in the cell membrane, gaseous ozone fumigation can efficiently kill bacteria [13,14]. The use of the ozonation process has a positive effect on reducing water losses during fruit storage, increases antioxidant activity, and reduces ethylene release by fruit subjected to the process [11,12,15]. The ozonation of the fruit’s surface may diminish ethylene secretion, and the treatment’s oxidative stress encourages the release of secondary metabolites. Due to its antibacterial properties and ability to preserve the fruit’s high nutritional and storage value, the use of ozone can raise the commercial worth of berries for the fresh market. Ozonation used in the appropriate dose changes metabolic processes, increasing the content of some bioactive compounds, and increasing the antioxidant value [15]. Phenylalanine ammonia lyase (PAL) is crucial in the creation of phenolic compounds within plants. Its primary function involves catalyzing the transformation of l-phenylalanine into trans-cinnamic acid. This acid can further be converted into various substances like flavonoids, phenolic acids, or anthocyanins. PAL tends to exhibit heightened activity within plant cells when subjected to diverse stressors like extreme temperature or humidity shifts, as well as exposure to UV radiation. Studies indicate a direct relationship between PAL activity and the abundance of phenolic compounds in plants [16]. Ozonation is an efficient postharvest preservation technique that increases fruit shelf life and minimizes the loss of nutrients, such as phenolic compounds, during storage. The profile of phenolic compounds and the amount of specific compounds may vary when ozone gas is used [15,16,17,18]. Elicitation is a stress factor that triggers the production of secondary metabolites, including phenolic compounds, antioxidants, and other metabolites. Ozonation may be a factor causing stress on the plant’s defense mechanisms, which stimulates the release of secondary metabolites and increases antioxidant properties. Ozonation as a method of elicitation might be applied at the stage of sowing seeds, planting, or during growth. Plant ozone defense responses may be different for the plant or raw material obtained [19]. The higher content of phenols in strawberry fruit after processing may be the result of ozone oxidative stress, which triggers plant defense mechanisms [20]. Moreover, the ozonation process also activates the enzymatic activity of phenylalanine ammonia lyase (PAL) and stimulates phenol production [21].

Highbush blueberry fruit has a shelf life of 10 to 18 days when stored properly at 0 °C and 90–95% humidity. Fresh blueberry fruit quality can be preserved during storage by using ozone in conjunction with an adequate chilling temperature [22,23]. In a study by Piechowiak et al. [15], the authors demonstrated that highbush blueberries’ high antioxidant capacity was sustained by using ozone treatment during storage, which also decreased anthocyanin, flavonoids, and ascorbic acid losses while minimizing grey mold symptoms (*Botrytis cinerea*). This research was carried out on well-known and commercially cultivated blueberry cultivars, such as “Bluecrop” and “Duke”.

Before new breeding clones of the highbush blueberry are released as cultivars and registered then introduced into cultivation, various studies must be conducted. This study aimed to assess the chemical properties in the fruits of six newest valuable Polish breeding clones and two highbush blueberry cultivars. The results of studies on the chemical properties of blueberry clones “BOR-9”, “-12”, “-17”, “-19”, “-20”, and “-21” have not yet been published. The effect of ozone fumigation on the polyphenolic profile and sugar content of the analyzed clones and cultivars during storage was also examined.

## 2. Results and Discussion

### 2.1. Identification of Phenolic Compounds

Highbush blueberries (*V. corymbosum* L.) have polyphenolic chemicals in their fruits, particularly anthocyanins, which have positive effects on health. These fruits enhance cardiovascular health [24], brain function, and memory [25], and they can regulate inflammation and lower oxidative stress [26]. The deterioration in cognitive and neural function brought on by aging and illnesses like Alzheimer’s can be treated with highbush blueberry fruit extract. The overall amount of bioactive substances that promote health is influenced by the cultivar, growing season, soil, and climate. Various storage and fruit processing procedures might lead to the degradation of anthocyanins and other polyphenols [5,6]. According to the state of knowledge, the degradation of phenolic compounds during fruit storage occurs as a result of enzymatic reactions caused by the action of oxidoreductase enzymes, mainly polyphenol oxidase. Ozone probably inactivates oxidizing enzymes, which slows down or completely inhibits the decomposition of phenolic compounds. In studies by Wahangchai et al. [27] and Barth et al. [28], it was observed that regardless of the time of exposure to ozone, the activity of polyphenol oxidase in stored fruit is inhibited. In turn, Ali et al. [29] suggested that the preventive effect of ozonation on the content of phenols results from the activation of enzymes responsible for the production of polyphenols in fruits (phenylalanine ammonia lyase, among others).

The highbush blueberry (*V. corymbosum* L.), which has various polyphenolic compound contents in its fruit, was studied for phenolic compounds using the UPLC-PDA-MS/MS method. This analysis allowed for researchers to identify differences in the contents of specific groups of polyphenolic compounds contained in the fruit of the analyzed cultivars and clones. Based on scientific findings [30,31,32], retention time (Rt), MS, and MS/MS were used to identify the chemicals. A total of 27 distinct substances were found in highbush blueberry fruit extracts, including 15 anthocyanins, 11 flavonoids, and 1 flavonic acid (Table A1, Table A2, Table A3, Table A4, Table A5, Table A6, Table A7 and Table A8 in Appendix A). Fruit ripeness (harvest date) and genotype both affected the overall concentration of phenolic chemicals. The genotypes of *V. corymbosum* L. that were examined in a study by Skrede et al. [9] had an anthocyanin profile that was distinct from our study’s findings. Comparing the findings of the two studies, the aforementioned researchers showed the presence of compounds like delphinidin-3-arabinoside and malvidin-3-arabinoside in the fruit of the genotypes tested, but they did not demonstrate the presence of compounds like Petunidin-3-O-(6″-acetyl)-glucoside or Cyanidin-3-O-(6″-malonyl)-galactoside in the fruit composition. Twenty-two distinct flavonoids, including those that were not found in the fruit of the genotypes examined in our investigation, such as Laricitrin 3-O-galactoside, Syringetin 3-O-galactoside, and Myricetin 3-O-galactoside, were identified by Kim et al. [33] in the composition of *V. corymbosum* fruit. The environmental factors present during plant vegetation as well as the selection of cultivars may have an impact on such substantial variations in the composition of the aforementioned chemicals in blueberry fruit (Table 1). Our investigation found a similar amount of anthocyanins in the fruit of *V. corymbosum* to those that had already been published [30,34]. Additionally, some of our genotypes’ fruit had amounts of individual flavonoids that were comparable to the findings of Kim et al. [33].

### 2.2. Anthocyanin Content

Anthocyanins, which are typical secondary metabolites, antioxidants, and pigments that contribute to the color of flowers and fruits, are a member of the polyphenols group. Although the amount of these chemicals varies from species to species, there are six major groups: pelargonidins, cyanidins, delphinidins, peonidins, and pelargonidins [9,35]. The majority of the phenolic compounds in the composition, or anthocyanins, account for on average 76.25% of all compounds identified in fruit. Malvidins make up 23% of anthocyanins, followed by petunidins at 16.5% and delphinidins at 15.11 % (Table A1, Table A2, Table A3, Table A4, Table A5, Table A6, Table A7 and Table A8). The content of the various anthocyanin groups in the fruit of the genotypes investigated in our investigation was equivalent to the findings of Wang et al. [30]. Out of the 15 different anthocyanins found in the fruit of the blueberry genotypes analyzed (Appendix A), 4 of them, namely, Malvidin-3-O-galactoside (M3Ga), Malvidin-3-O-glucoside (M3Gl), Delphinidin-3-O-glucoside (D3G), and Cyanidin-3-O-glucoside (C3G), exhibited the highest content irrespective of the genotype. The content of these four anthocyanins is presented in Table 1. The fresh fruit with the highest anthocyanin content, of 105.63 mg·100 g^−1^ f.w., came from the BOR-21 clone and had not been ozone-treated (Table A6). The fruits of clone BOR-17, on the other hand, had the lowest anthocyanin concentration (44.59 mg·100 g^−1^ f.w.) after being ozonated for 15 min at a dose of 0.01 mL·L^−1^ (Table A3). After one day of storage, the blueberry fruits that were not treated with gaseous ozone showed a higher D3G content for clones BOR-9, 12, 17, 21, and the “Bluecrop” and “Duke” cultivars. However, there was a lower D3G content for clones 19 and 20. With regard to C3G anthocyanins, except for clones 9, 19, and 21, the fruits treated with ozone for 30 min showed a higher C3G content compared to the control sample, which consisted of untreated fruits. Except for clones 9, 21, and the “Bluecrop” variety for M3CL and clones 9, 17, and 19 for M3GA, the ozonated fruit had higher concentrations of the tested compounds. This relationship was observed similarly for M3CL and M3GA. Compared to trials ozonated with doses of 0.01 mL·L^−1^ for 15 min and 0.01 mL·L^−1^ for 30 min., respectively, an average of 16.95% and 14.31% more of this chemical was discovered in the fruit of the control experiment. Fruits of clone BOR-21 were characterized by the highest contents of M3Ga—12.02 mg·100 g^−1^ f.w., M3Gl—16.32 mg·100 g^−1^ f.w., D3G—14.80 mg·100 g^−1^ f.w., and C3G—12.18 mg·100 g^−1^ f.w. (Table A6).

Fruits from the tested clones and cultivars that had been stored for 7 days and ozonated for 30 min at a level of 0.01 mL·L^−1^ were distinguished from the control sample by having a higher anthocyanin content. The fruit of the clone BOR-9 has the highest amount of this natural pigment—120.40 mg·100 g^−1^ f.w. (Table A1). Fruit from the investigated genotypes ozonated for 30 min at a dosage of 0.01 mL·L^−1^ on average contained 34% more anthocyanins than the control sample. The amount of M3Ga, M3Gl, D3G, and C3G in fruit from the examined cultivars and clones that had been stored for 7 days was significantly higher in samples that had been ozonized for 30 min at a dose of 0.01 mL·L^−1^. The fruit of clone BOR-9 had the highest concentration of the aforementioned chemicals. (Table A1). The control sample’s M3Ga level was only higher in the fruit of the cultivar “Duke” and clone BOR-17 (Table A3 and Table A8).

Anthocyanin content in fruit that had been ozonated for 30 min after being stored for 14 days was found to be 20.53% and 17.80% higher than in the control and sample that had only been exposed to 0.01 mL·L^−1^ of ozone for 15 min, respectively. Clone BOR-21 had the highest anthocyanin content—124.33 mg·100 g^−1^ f.w. (Table A6). In comparison to the control sample, ozonated fruit had increased levels of M3Ga, M3Gl, D3G, and C3G after 14 days of storage (Table A1, Table A2, Table A3, Table A4, Table A5, Table A6, Table A7 and Table A8). A study by Wang et al. [30] found that 12 cultivars out of 62 evaluated had anthocyanin contents in the range of 0 to 200 mg·100 g^−1^ f.w., which was comparable to the total anthocyanin content in the fruit of the genotypes of highbush blueberries under analysis. Anthocyanins are unstable substances, particularly following an extraction procedure. Temperature, storage time, pH level, oxygen content, enzyme presence, and ascorbic acid all had an impact on how stable this chemical was in the fruit [34]. Because anthocyanins have health-promoting qualities as well as sensory appeal, it is crucial to maintain a high level of them in fruits. In an experiment by Sachadyń-Król [19], scientists used ozonation to increase the anthocyanin content of *V. corymbosum* fruits. According to studies [36,37], after exposure to ozone gas, strawberries, and raspberries exhibited a greater anthocyanin concentration.

### 2.3. Content of Other Phenolic Compounds

Strong antioxidant properties of flavonoids and phenolic acids prevent DNA, lipid, and protein oxidation, which can cause cancer, diabetes, and disorders of the liver, blood vessels, and brain. Depending on the variety, growing environment, and season, various chemicals may be present in varying amounts. One of the phenolic acids found in foods most frequently is chlorogenic acid, which has potent antioxidant qualities. It regulates metabolic diseases and has an impact on lipid and glucose metabolism [3,31]. In the fruit of the highbush blueberry genotypes examined, flavonoids and chlorogenic acid (CGA) made for 23.75% of all phenolic compounds found (Table A1, Table A2, Table A3, Table A4, Table A5, Table A6, Table A7 and Table A8). No matter how long the fruit was stored or how much ozone was used, the average content of these compounds was 72.09% CGA, 24.59% Quercetin compounds, and traces of Myricetin 3-O-glucoside and Iso-rhamnetin 3-O-rutinoside (Table A1, Table A2, Table A3, Table A4, Table A5, Table A6, Table A7 and Table A8). During the 7 days of storage, the fruit of clone BOR-21 in the control sample had the highest concentration of other phenolic compounds—27.54 mg·100 g^−1^ f.w. (Table A6). The “Duke” cultivar of fruit that had been ozonated at a dose of 0.01 mL·L^−1^ for 15 min had the lowest concentration (17.52 mg·100 g^−1^ f.w.) of these chemicals. Findings of Sun et al. [38], in which the researchers estimated around 8.0 mg·100 g^−1^ f.w. of flavonoids in ripe fruit, were equivalent to the flavonoid content of the fruit of the genotypes studied. The cultivar “Duke,′′ whose fruit was ozonized with a dose of 0.01 mL·L^−1^ for 15 min, had the lowest level (17.52 mg·100 g^−1^ f.w.) of other phenolic compounds in fresh fruit, while the cultivar “Bluecrop” had the highest content (26.33 mg·100 g^−1^ f.w.). Only the clones BOR-9 and BOR-20 and the cultivar “Duke” showed an increase in flavonoid and chlorogenic acid content in fresh fruit ozonated with both doses (0.01 mL·L^−1^ for 15 min and 0.01 mL·L^−1^ for 30 min) (Table A1, Table A5 and Table A8).

Following the application of a dose of 0.01 mL·L^−1^ for 15 min to fruit that had been stored for 7 days, an increase in the remaining phenolic compounds was observed for the clones BOR-17 and BOR-19 (Table A3 and Table A4), as well as for the clones BOR-9 and BOR-20 and cultivar “Duke” (Table A1, Table A5 and Table A8). The fruit of clone BOR-17, which was ozonized at a dose of 0.01 mL·L^−1^ for 15 min, contained the largest amount of these compounds (26.45 mg·100 g^−1^ f.w.), whereas clone BOR-9, whose fruit was not ozonized, had the lowest amount (18.43 mg·100 g^−1^ f.w.). In comparison to the control sample, fruits that were ozonized for 30 min at a dose of 0.01 mL·L^−1^ had on average 8.1% higher CGA content (Table A1, Table A2, Table A3, Table A4, Table A5, Table A6, Table A7 and Table A8). The fruit of the clone BOR-21 ozonized at a dose of 0.01 mL·L^−1^ for 30 min had the highest concentration of CGA (18.96 mg·100 g^−1^ f.w.) (Table A6).

In our investigation, fruit from the highbush blueberry clones BOR-12, BOR-20, and BOR-21 contained more flavonoids and chlorogenic acid after 14 days of storage compared to the control (Table A2, Table A3, Table A4, Table A5 and Table A6). In the fruit that was not treated with ozone, the other cultivars and clones that were evaluated contained more of the other phenolic compounds. The fruit of clone BOR-20 without ozonation had the highest concentration (25.32 mg·100 g^−1^ f.w.) and the lowest concentration (17.08 mg·100 g^−1^ f.w.) of these chemicals, respectively (Table A5 and Table A6). With fruit treated with ozonation of 0.01 mL·L^−1^ for 30 min (Table A1, Table A2, Table A3, Table A4, Table A5, Table A6, Table A7 and Table A8), the genotypes evaluated had somewhat greater average CGA content. The genotypes found in our investigation had CGA content in their fruit that was comparable to findings of Skrede et al. [9]. Ozonation alters the skin’s and cells’ cell walls, which could lead to a rise in the concentration of phenolic chemicals. Phenolic compounds can build up in cell walls depending on the structure. Ozone, which raises their content, causes changes in the concentration of phenolic compounds and their activity. In research by Sachadyń-Król [19], phenolic content in *V. corymbosum* increased following the use of gaseous ozone. Using the right amount of ozone, a study by [36,37] found that the phenolic content of strawberries and raspberries was increased.

### 2.4. Total Phenolic Compounds

The range of total phenolic compounds (TPC) in the fruit of the genotypes of highbush blueberries under study ranged from 62.69 to 144.80 mg·100 g^−1^ f.w. (Table A1, Table A2, Table A3, Table A4, Table A5, Table A6, Table A7 and Table A8). The fruit from clone BOR-9 had the highest TPC content (144.80 mg·100 g^−1^ f.w.), which was discovered on the seventh day of storage at an ozone dose of 0.01 mL·L^−1^ for 30 min (Table A1), whereas fresh fruit from clone BOR-17 had the lowest TPC (62.69 mg·100 g^−1^ f.w.) at the same ozone dose (0.01 mL·L^−1^ for 15 min) (Table A3). Twenty-two out of the seventy-two fruit samples examined (replicates) showed phenolic component levels (TPC) above 100 mg·100 g^−1^ f.w., with 68.2% of these samples being ozone-treated fruit. Only the fruit of two clones, BOR-9 and BOR-20, and the cultivar “Bluecrop” had a higher phenolic content in ozonated samples in fresh fruit; there was no discernible effect of ozonation treatment on the saturation of other phenolic compounds (Table A1, Table A2, Table A3, Table A4, Table A5, Table A6, Table A7 and Table A8).

Fruit from the investigated genotypes that had been kept for 7 days had phenolic compounds in the range from 73.52 to 144.80 mg·100 g^−1^ f.w. Following seven days of storage, fruit treated with 0.01 mL·L^−1^ of ozone increased in phenolic compound content in both breeding clones and cultivars. Comparing fruit that had received the aforementioned amount of ozonation to control samples, the average level of these chemicals across all genotypes was 28.60% higher (Table A1, Table A2, Table A3, Table A4, Table A5, Table A6, Table A7 and Table A8).

Fruit that had been preserved for 14 days contained phenolic chemicals in amounts between 73.41 and 143.73 mg·100 g^−1^ f.w. On day 14 of storage, fruit treated with 0.01 mL·L^−1^ of ozone for 30 min had a 16.50% increase in overall average phenolic component concentration compared to control samples. On day 14 of storage, only a 3% rise in phenolic component content was observed when a dose of 0.01 mL·L^−1^ of ozone was administered for 15 min (Table A1, Table A2, Table A3, Table A4, Table A5, Table A6, Table A7 and Table A8). The results of other research were equivalent to what we found for the overall amount of phenolic chemicals in the fruit of the genotypes we tested [30,34]. Average statistics show that ozonated fruits have a higher quantity of bioactive chemicals than non-ozonated fruits. However, individual results depend on the type and length of ozonation. This has to do with how metabolic processes that raise the concentration of chemicals that promote health are affected by the oxidative stress of ozonation. Because ozonation increases the average concentration of D3G, C3G, M3GL, M3GA, P3G, and CHA in fruits, lowering the release of ethylene from berries may result in a slower rate of bioactive component breakdown and ripening of fruit [13,34]. The ozonation process has an effect on the raw material’s surface, and in the case of blueberries, it has an effect on the tissue that can store secondary metabolites. Oxidative stress results in an increase in the concentration of particular chemicals. [39].

Many studies have shown that at specific doses, ozone causes the accumulation of reactive oxygen species (ROS) in fruits and vegetables as a natural response to abiotic stress factors [12,23,40]. As a result of accumulating ROS, plant organs induce the synthesis of phytoalexins (isoflavonoids, stilbenephytoalexins) and other antioxidant and antimicrobial systems. There is little research on explaining the effect of ozone gas on berries [12,22]. According to Sachadyń–Król [19], elicitation caused by abiotic stress improved biological activity of *V. corymbosum*, making this raw material biologically better.

### 2.5. Content of Sugars

Regardless of storage period or ozone exposure, the examined highbush blueberry genotypes’ tested fruits had a total sugar content that ranged from 13.74 to 23.57 g·100 g^−1^, with 6.54 to 11.91 g·100 g^−1^ of fructose and 7.20 to 11.66 g·100 g^−1^ of glucose (Table 2).

The results obtained by Kraśniewska et al. [41] were comparable to our findings regarding the concentration of total sugars, fructose, and glucose in the fruit of the genotypes of *V. corymbosum* evaluated in our investigation. The fruit was fresh and not kept, and the clone BOR-12, which was ozonated for 30 min at a dose of 0.01 mL·L^−1^, had the highest sugar content (22.39 g·100 g^−1^) (Table 2). Fruit from clone BOR-20 had the lowest concentration (15.41 g·100 g^−1^) of total sugars after receiving an ozone exposure of 0.01 mL·L^−1^ for 15 min (Table 2). Comparing fruit samples after one day of storage and treated with 0.01 mL·L^−1^ of ozonation for 15 and 30 min to control samples, it was found that the sugar content was higher in the most of the ozonation-treated fruit samples (less content found in BOR-9, BOR-19, and BOR-21). In the BOR-9 control sample, total sugar content was 20.00 g·100 g^−1^, while in fruits exposed to ozonation for 30 min, sugar content was 18.71 g·100 g^−1^. In the BOR-17 control sample, total sugar content was 19.29 g·100 g^−1^, while in fruits exposed to ozonation for 30 min. sugar content was 17.77 g·100 g^−1^. In the BOR-21 control sample, total sugar content was 16.80 g·100 g^−1^ and 16.40 g·100 g^−1^ in the sample treated with ozone. Fruit ozonized with a dose of 0.01 mL·L^−1^ for 30 min had an average sugar content that was 4.4% greater than the control sample. The most successful ozonation method for fresh fruit was the one that lasted 30 min (Table 2). The studied cultivars and clones’ total sugar content in fruit that was kept for 7 days varied from 13.74 to 22.74 g·100 g^−1^. Comparing the ozonized samples to the control samples, the average amount of total sugars in the fruit of the examined cultivars and clones of *V. corymbosum* was 6.4% greater. Fruit from the investigated genotypes that had been preserved for 7 days ranged in fructose content from 6.54 to 11.31 g·100 g^−1^. The amount of glucose was between 7.20 and 11.43 g·100 g^−1^ (Table 2). The ozonation process that lasted for 15 min was the most successful for fruit that was kept for 7 days but adding another 15 min to the ozonation process also reduced the amount of sugar in the blueberry fruit (Table 2).

The total sugar content of blueberry fruit stored for 14 days ranged from 15.26 to 23.57 g·100 g^−1^ (Table 2). Fruits exposed to ozonation for 30 min at a concentration of 0.01 mL·L^−1^ had an average sugar content that was 6.3% higher than control samples. Only fruit from the clones BOR-20 and BOR-21 displayed a higher total sugar content in the control sample when compared to fruit that had undergone ozonation. Fruit that had been kept in storage for 14 days ranged in fructose content from 7.57 to 11.91 g·100 g^−1^ and in glucose content from 7.84 to 11.66 g·100 g^−1^ (Table 2). Ozonation for 30 min was the most efficient procedure for blueberry fruit that had been refrigerated for 14 days. The higher respiration rate of berries, which may also be brought on by the use of an excessive dosage of ozone, may be the source of the reduced sugar concentration in the fruit in the control sample. To obtain the best possible results from the treatment, the right ozone dose must be determined. In a study by Głowacz [42], ozonation of bell peppers at a dose of 0.1 µmol^.^ mol^−1^ increased the total sugar content, but at a level of 0.3 µmol^.^ mol^−1^, the amount of all sugars was decreased. Ozone exposure at a level of 4 µmol^.^ mol^−1^ every three hours preserved tomato sugars according to a study by Aguayo [43].

The effect of ozonation on the chemical properties (polyphenol content, antioxidant value) and mechanical properties of the fruit during storage was studied, but due to the large number of results obtained, these will be presented in the next article.

## 3. Material and Methods

### 3.1. Material

Fruits from newly selected Polish highbush blueberry clones (northern type, numbered “BOR-9”, “-12”, “-17”, “-19”, “-20”, and “-21”) and two cultivars commonly grown commercially—”Bluecrop” and “Duke”—were used as study material. These clones are the result of applied breeding of this species carried out at the Department of Horticultural Crop Breeding since 2009 and financed by the Ministry of Agriculture and Rural Development in Warsaw. Fruits were harvested by hand from bushes of the above genotypes grown in a trail in the field of the Pomological Orchard in Skierniewice (51°57′38′’ N, 20°8′39′’ E, Łódzkie Voivodeship, central Poland), belonging to the National Institute of Horticultural Research (InHort) in Skierniewice, in 2022. Fruits were harvested when they were fully ripe for harvesting, depending on color, flavor, and the strength of the attachment of the fruit to the stalk. Freshly harvested fruit samples were stored at room temperature (4 °C) for 14 days.

### 3.2. Ozone Treatment of Fruit

Fruit from the blueberry cultivars and clones analyzed were randomly divided into three batches shortly after harvest. Fruit that had not been ozonated was used as a control sample in the first batch. The two further batches underwent ozonation treatment in plastic containers. With a gaseous ozone flow of 40 g O_3_^.^h^−1^*,* a gaseous ozone concentration of 0.01 mL·L^−1^ was delivered for 15 min to the second batch and 30 min to the third batch. Gaseous ozone was produced using a KORONA A 40 Standard generator (Korona, Piotrków Trybunalski, Poland), and its quantity was measured using an Ozone Solution 106 M UV detector (Hull, MA, USA).

### 3.3. Sample Preparation

Ten grams of blueberry fruits were homogenized with 50 mL of 50% MeOH in water using a homogenizer T 25 Ultra Turrax (IKA, Warsaw, Poland) for chromatographic analysis.

The homogenate was transferred into Falcon tubes of 50 mL capacity and centrifuged at 7500 rpm for ten min using a Centrifuge 5430 (Eppendorf AG, Hamburg, Germany). Before injection, the supernatants were diluted with mobile phase 1:4 (v/v). The UPLC-PDA-MS method was employed for the identification of particular polyphenolic compounds.

### 3.4. Identification and Quantification of Polyphenols in Extract by the UPLC-PDA-MS Method

The polyphenolic profile of blueberries was evaluated using Kapusta et al.’s [44] methodology. By using the ultraperformance liquid chromatography combined with a photodiode array and tandem quadrupole mass detector with electrospray ionization (UPLC-PDA-TQD) ACQUITY system (Waters, Milford, MA, USA), the individual polyphenolic chemicals were identified and quantified. On a BEH C18 column (100 mm × 2.1 mm i.d., 1.7 μm, Waters), chromatographic separation was carried out. The mobile phase was MILLI-Q water with 0.1 formic acid (component A) and 40% ACN in water with 0.1% formic acid (component B). The investigation of anthocyanins utilized the subsequent solvent system: mobile phase A (2% formic acid in water *v*/*v*) and mobile phase B (2% formic acid in 40% ACN in water *v*/*v*). The gradient program was established as follows: at 0 min, 5% B was used, linearly increasing to 100% B from 0 to 8 min; then, a washing stage was carried out from 8 to 9.5 min, returning to the initial conditions afterwards. The temperature of the column was set at 50 °C. The injected sample volume was 5 µL (partial loop with needle overfill). For the mass spectrometer the following parameters were used: capillary voltage, 3.5 kV; con voltage, 30 V in negative mode; the source was kept at 120 °C and the desolvation temperature was 350 °C; con gas flow, 100 L/h; and desolvation gas flow, 800 L/h. Argon was utilized as the collision gas at a flow rate of 0.3 mL/min. To identify peaks, a particular PDA spectra, mass-to-charge ratio, and fragment ions obtained after collision-induced dissociation (CID) of the samples were compared to those of the standard substances. Additionally, a comparison with literature data was conducted. The identified compounds were quantified based on multiple point calibration curves with solution ranging from 0.05 to 5 mg/mL (R^2^ ≤ 0.9998) of phenolic compounds as standards. Each experiment was performed with three parallels and the average value was taken. Waters MassLynx software v.4.1 was used for data acquisition and processing.

### 3.5. Determination of Sugars

The sugar concentration was determined using the HPLC method with refractive index detection. The chromatographic apparatus Sykam (Sykam GmbH, Eresing, Germany), which includes the sample injector S5250, pump system S1125, column oven S4120, and RI detector S3590, was employed. The Cosmosil Sugar-D column (Nacalai Tesque, Kyoto, Japan) of dimensions 5 µm, 4.6 mm × 250 mm was used for separation. Seventy percent ACN in water was used as the mobile phase in an isocratic method to produce the separation. The column temperature was set at 30 °C, and the flow rate was 0.5 mL/min. A sample volume of 20 µL was injected, and the analysis was completed within 20 min. Every determination was conducted thrice. Identification of compounds was accomplished by comparing the retention time with real standards.

### 3.6. Statistical Analysis

Statistical analysis was performed using STATISTICA 13.3 software from TIBCO Software Inc, Tulsa, Oklahoma, USA. The two way analysis of variance (ANOVA) and LSD significance test were used with a significance level of α = 0.05. The Tukey test was used for statistical testing of the research results.

## 4. Conclusions

The amount of phenolic compounds and sugars in the fruit of the investigated genotypes of highbush blueberry (*V. corymbosum* L.) was positively impacted by the application of ozone gas during storage. In blueberry fruit, M3Ga, M3Gl, D3G, C3G, and P3GL were the primary anthocyanins. The findings suggest that treating fruit with ozone for 30 min increased the amount of anthocyanin in comparison to the control sample when stored for 14 days. Furthermore, in all fruit (except clone BOR-17), the level of anthocyanin in the ozonated fruit after 14 days of storage was higher than that of the fresh fruit. CGA was the most prevalent of the other phenolic compounds. The average amount of phenolic compounds in the fruit of all cultivars and clones studied increased by 28.60% on average after 7 days of storage, with anthocyanins accounting for 34% of this rise. The fruit of the investigated genotypes displayed an average rise in phenolic components of 16.50% after 14 days of storage, including anthocyanins of 20.53%. On days 7 and 14 of storage, fruit that had been ozonized with a dose of 0.01 mL·L^−1^ for 30 min had an average total sugar content that was 6.4% and 6.3% greater. For all tested clones and cultivars (except for BOR-20 and BOR-21), the application of ozone for 30 min in fruit stored for 14 days exhibited an augmentation in sugar content. A substantially greater quality of fruit may result from further research into the use of gaseous ozone in the storage of blueberry fruit. Fruit’s composition in health-promoting chemicals is positively impacted by the application of ozonation treatments. Further research may be required due to potential difficulties in creating an efficient technology and calculating ozone gas doses.

## Figures and Tables

**Table 1:** Total content of selected compounds in the fruit of the studied highbush blueberry genotypes.

Variety	Storage Day	Ozone Exposure Time (min)	D3G (mg·100 g^−1^ f.w)	C3G (mg·100 g^−1^ f.w)	M3GL (mg·100 g^−1^ f.w)	M3GA (mg·100 g^−1^ f.w)	P3GL mg·100 g^−1^ f.w)	Total Anthocyanins (mg·100 g^−1^ f.w)	CGA (mg·100 g^−1^ f.w)	Q3Gl (mg·100 g^−1^ f.w)	Q3Ga (mg·100 g^−1^ f.w)	Total Other Phenolics (mg·100 g^−1^ f.w)
Clone BOR–9	1	0	6.43 ^b^ ± 0.08	5.83 ^a^ ± 0.33	8.86 ^a^ ± 0.37	7.23 ^ab^ ± 0.41	6.21 ^a^ ± 0.27	34.56	16.96 ^f^ ± 0.37	1.35 ^a^ ± 0.18	0.81 ^a^ ± 0.15	19.12
		30	6.36 ^b^ ± 0.04	5.88 ^a^ ± 0.12	9.43 ^b^ ± 0.07	7.48 ^b^ ± 0.16	6.64 ^c^ ± 0.13	35.79	15.54 ^c^ ± 0.21	1.58 ^b c^ ± 0.17	0.89 ^ab^ ± 0.28	18.01
	14	0	7.44 ^c^ ± 0.16	6.43 ^c^ ± 0.09	10.47 ^c^ ± 0.42	9.69 ^d^ ± 0.08	6.29 ^a^ ± 0.21	40.32	16.22 ^d^ ± 0.25	2.50 ^f^ ± 0.21	1.68 ^d^ ± 0.27	20.40
		30	10.97 ^f^ ± 0.31	10.01 ^d^ ± 0.41	11.47 ^c^ ± 0.24	8.91 ^c^ ± 0.16	8.46 ^e^ ± 0.15	49.82	16.66 ^e^ ± 0.31	1.70 ^c^ ± 0.20	1.20 ^c^ ± 0.11	19.56
Clone BOR- 12	1	0	9.17 ^e^ ± 0.22	8.09 ^e^ ± 0.16	13.86 ^d^ ± 0.18	9.47 ^d^ ± 0.11	9.35 ^f^ ± 0.21	49.94	17.13 ^e^ ± 0.34	2.19 ^cd^ ± 0.38	1.47 ^c^ ± 0.22	20.79
		30	4.89 ^b^ ± 0.21	4.96 ^a^ ± 0.16	11.10 ^a^ ± 0.02	9.52 ^de^ ± 0.13	7.82 ^c^ ± 0.12	38.29	16.51 ^d^ ± 0.31	2.12 ^c^ ± 0.03	1.13 ^b^ ± 0.06	19.76
	14	0	7.99 ^d^ ± 0.09	7.32 ^d^ ± 0.16	14.47 ^e^ ± 0.34	10.62 ^f^ ± 0.29	8.00 ^d^ ± 0.10	48.40	15.59 ^b^ ± 0.25	1.91 ^c^ ± 0.15	0.93 ^ab^ ± 0.19	18.43
		30	14.99 ^g^ ± 0.33	11.54 ^g^ ± 0.32	13.36 ^c^ ± 0.17	9.33 ^d^ ± 0.06	8.28 ^e^ ± 0.06	57.50	15.97 ^c^ ± 0.05	2.93 ^e^ ± 0.21	1.78 ^d^ ± 0.12	20.68
Clone BOR–17	1	0	12.89 ^h^ ± 0.09	10.02 ^g^ ± 0.34	16.09 ^g^ ± 0.16	11.64 ^g^ ± 0.29	9.83 ^h^ ± 0.18	60.47	15.12 ^a^ ± 0.35	1.93 ^bc^ ± 0.11	0.82 ^b^ ± 0.18	17.87
		30	7.66 ^d^ ± 0.14	6.43 ^d^ ± 0.12	10.99 ^b^ ± 0.21	9.10 ^c^ ± 0.21	6.13 ^b^ ± 0.03	40.31	15.78 ^b^ ± 0.37	1.72 ^b^ ± 0.21	0.86 ^b^ ± 0.12	18.36
	14	0	4.87 ^b^ ± 0.11	5.20 ^c^ ± 0.16	10.07 ^a^ ± 0.11	9.68 ^d^ ± 0.10	6.82 ^c^ ± 0.25	36.64	16.29 ^c^ ± 0.32	1.90 ^b^ ± 0.23	1.12 ^c^ ± 0.28	19.31
		30	9.64 ^f^ ± 0.22	7.69 ^f^ ± 0.14	11.49 ^c^ ± 0.01	10.87 ^e^ ± 0.11	7.74 ^e^ ± 0.08	47.43	15.86 ^b^ ± 0.31	2.28 ^c^ ± 0.32	1.12 ^c^ ± 0.18	19.26
Clone BOR–19	1	0	10.30 ^d^ ± 0.22	8.78 ^e^ ± 0.21	11.83 ^c^ ± 0.22	10.60 ^f^ ± 0.29	7.91 ^d^ ± 0.17	49.42	16.00 ^b^ ± 0.34	1.49 ^a^ ± 0.24	0.97 ^ab^ ± 0.08	18.46
		30	12.03 ^f^ ± 0.22	9.65 ^g^ ± 0.08	14.14 ^f^ ± 0.25	7.19 ^b^ ± 0.24	7.72 ^d^ ± 0.07	50.73	15.75 ^b^ ± 0.31	2.23 ^c^ ± 0.25	1.08 ^b^ ± 0.16	19.06
	14	0	11.43 ^e^ ± 0.21	9.15 ^f^ ± 0.11	12.39 ^d^ ± 0.21	9.20 ^e^ ± 0.10	7.26 ^c^ ± 0.31	49.43	15.88 ^b^ ± 0.12	1.97 ^b^ ± 0.25	1.51 ^c^ ± 0.31	19.36
		30	14.24 ^g^ ± 0.11	11.53 ^h^ ± 0.08	14.48 ^f^ ± 0.21	8.89 ^d^ ± 0.04	9.81 ^f^ ± 0.31	58.95	17.86 ^d^ ± 0.33	1.94 ^b^ ± 0.07	1.47 ^c^ ± 0.21	21.27
Clone BOR–20	1	0	7.84 ^c^ ± 0.22	6.39 ^b^ ± 0.18	9.34 ^a^ ± 0.25	8.75 ^d^ ± 0.09	6.57 ^b^ ± 0.14	38.89	14.75 ^a^ ± 0.29	1.26 ^a^ ± 0.12	0.73 ^a^ ± 0.08	16.74
		30	7.90 ^c^ ± 0.10	7.01 ^c^ ± 0.31	11.63 ^e^ ± 0.26	9.34 ^e^ ± 0.13	6.23 ^a^ ± 0.16	42.11	15.67 ^c^ ± 0.21	1.70 ^bc^ ± 0.10	0.87 ^a^ ± 0.15	18.24
	14	0	9.22 ^e^ ± 0.13	8.20 ^d^ ± 0.15	12.66 ^g^ ± 0.32	10.23 ^g^ ± 0.14	8.12 ^e^ ± 0.19	48.43	17.07 ^d^ ± 0.33	2.27 ^d^ ± 0.25	1.41 ^b^ ± 0.24	20.75
		30	10.58 ^f^ ± 0.09	9.03 ^e^ ± 0.16	11.60 ^e^ ± 0.10	8.47 ^c^ ± 0.14	9.04 ^f^ ± 0.09	48.72	17.57 ^e^ ± 0.25	2.53 ^e^ ± 0.28	0.92 ^a^ ± 0.19	21.02
Clone BOR–21	1	0	14.80 ^g^ ± 0.26	12.18 ^f^ ± 0.15	16.32 ^g^ ± 0.27	12.02 ^f^ ± 0.25	10.38 ^e^ ± 0.11	65.70	16.27 ^d^ ± 0.34	2.40 ^c^ ± 0.18	1.29 ^bc^ ± 0.30	19.96
		30	8.52 ^d^ ± 0.22	6.80 ^c^ ± 0.13	10.49 ^b^ ± 0.31	8.14 ^b^ ± 0.14	6.11 ^a^ ± 0.09	40.06	15.68 ^c^ ± 0.29	1.71 ^b^ ± 0.15	0.79 ^a^ ± 0.08	18.18
	14	0	9.04 ^e^ ± 0.21	7.72 ^d^ ± 0.15	11.37 ^c^ ± 0.36	6.67 ^a^ ± 0.24	6.96 ^b^ ± 0.21	41.76	12.85 ^a^ ± 0.15	0.91 ^a^ ± 0.13	1.15 ^b^ ± 0.19	14.91
		30	14.22 ^f^ ± 0.31	11.74 ^e^ ± 0.25	26.51 ^h^ ± 0.36	8.19 ^b^ ± 0.16	20.35 ^f^ ± 0.11	81.01	15.28 ^b^ ± 0.33	1.16 ^a^ ± 0.11	0.59 ^a^ ± 0.17	17.03
“Bluecrop”	1	0	7.44 ^b^ ± 0.15	6.69 ^c^ ± 0.27	12.28 ^b^ ± 0.17	9.40 ^c^ ± 0.11	8.55 ^d^ ± 0.16	44.36	18.79 ^e^ ± 0.24	2.55 ^b^ ± 0.21	1.73 ^c^ ± 0.13	23.07
		30	7.42 ^b^ ± 0.13	6.33 ^b^ ± 0.23	10.75 ^a^ ± 0.19	8.12 ^b^ ± 0.12	7.32 ^c^ ± 0.22	39.94	16.10 ^a^ ± 0.28	2.28 ^b^ ± 0.08	1.39 ^bc^ ± 0.12	19.77
	14	0	6.18 ^a^ ± 0.13	5.77 ^a^ ± 0.09	10.86 ^a^ ± 0.16	8.30 ^b^ ± 0.10	7.67 ^c^ ± 0.17	38.78	17.87 ^d^ ± 0.16	2.35 ^b^ ± 0.03	1.57 ^c^ ± 0.14	21.79
		30	11.66 ^e^ ± 0.14	10.75 ^e^ ± 0.31	13.30 ^c^ ± 0.10	12.46 ^e^ ± 0.01	10.29 ^g^ ± 0.06	58.46	16.15 ^a^ ± 0.34	2.69 ^b^ ± 0.13	1.63 ^c^ ± 0.11	20.47
“Duke”	1	0	11.30 ^d^ ± 0.15	7.92 ^c^ ± 0.10	11.68 ^d^ ± 0.11	8.24 ^d^ ± 0.14	6.13 ^b^ ± 0.08	45.27	14.39 ^b^ ± 0.23	0.98 ^a^ ± 0.22	0.66 ^a^ ± 0.13	16.03
		30	6.62 ^b^ ± 0.07	5.71 ^b^ ± 0.10	12.73 ^f^ ± 0.13	10.00 ^f^ ± 0.11	7.62 ^e^ ± 0.22	42.68	15.28 ^c^ ± 0.18	2.18 ^c d^ ± 0.13	1.44 ^c^ ± 0.12	18.90
	14	0	14.22 ^g^ ± 0.12	11.12 ^d^ ± 0.14	13.41 ^g^ ± 0.02	7.96 ^c^ ± 0.08	7.16 ^d^ ± 0.10	53.87	16.69 ^f^ ± 0.21	1.91 ^c^ ± 0.11	1.44 ^c^ ± 0.08	20.04
		30	13.12 ^f^ ± 0.02	11.32 ^d^ ± 0.08	13.88 ^h^ ± 0.21	9.60 ^e^ ± 0.05	9.43 ^g^ ± 0.01	57.35	16.38 ^e^ ± 0.15	1.95 ^c^ ± 0.15	1.23 ^b^ ± 0.10	19.56
Average	1	0	10.02	8.24	11.84	9.35	8.12	47.57	16.38	1.83	1.09	19.30
		30	7.67	6.60	10.48	7.93	6.95	39.63	15.52	1.82	0.93	18.27
	14	0	8.80	7.61	11.26	8.93	7.29	43.89	16.27	2.03	1.38	19.68
		30	12.42	10.45	13.72	9.26	10.43	56.28	16.51	2.13	1.22	19.86

Data are expressed as mean values (*n* = 3) ± SD; SD—standard deviation. Mean values within columns with different letters are significantly different (*p* < 0.05) according to the Tukey test. Delphinidin-3-O-glucoside (D3G), Cyanidin-3-O-glucoside (C3G), Malvidin-3-O-glucoside (M3Gl), Malvidin-3-O-galactoside (M3Ga), Petunidin-3-O-glucoside (P3GL), Chlorogenic acid (CGA), Quercetin 3-O-glucoside (Q3Gl), Quercetin 3-O-galactoside (Q3Ga).

**Table 2:** Total sugar content in the fruit of the studied highbush blueberry genotypes.

Clone/Cultivar	Storage Time (days)	Ozone Exposure Time (min)	Fructose Content (g·100 g^−1^)	Glucose Content (g·100 g^−1^)	Total Sugar Content (g·100 g^−1^)
Clone BOR-9	1	0	10.09 ^g^ ± 0.14	9.91 ^f^ ± 0.13	20.00 ^i^ ± 0.27
		15	9.34 ^ef^ ± 0.34	9.34 ^de^ ± 0.34	18.67 ^gh^ ± 0.67
		30	8.99 ^e^ ± 0.11	9.72 ^ef^ ± 0.12	18.71 ^gh^ ± 0.23
	7	0	9.75 ^fg^ ± 0.15	9.38 ^e^ ± 0.15	19.13 ^h^ ± 0.30
		15	9.06 ^e^ ± 0.16	8.82 ^cd^ ± 0.23	17.88 ^g^ ± 0.12
		30	9.78 ^fg^ ± 0.22	9.41 ^e^ ± 0.11	19.19 ^h^ ± 0.18
	14	0	7.57 ^b^ ± 0.09	8.66 ^c^ ± 0.03	16.23 ^d^ ± 0.10
		15	8.39 ^d^ ± 0.08	9.09 ^d^ ± 0.16	17.48 ^fg^ ± 0.14
		30	10.16 ^g^ ± 0.06	9.56 ^e^ ± 0.22	19.72 ^hi^ ± 0.09
Clone BOR-12	1	0	9.43 ^f^ ± 0.11	9.56 ^e^ ± 0.11	18.99 ^h^ ± 0.07
		15	8.34 ^d^ ± 0.17	9.65 ^e^ ± 0.14	17.99 ^g^ ± 0.13
		30	10.80 ^h^ ± 0.13	11.59 ^i^ ± 0.23	22.39 ^k^ ± 0.19
	7	0	7.65 ^b^ ± 0.22	8.85 ^cd^ ± 0.27	16.50 ^e^ ± 0.23
		15	7.33 ^b^ ± 0.26	9.11 ^d^ ± 0.08	16.44 ^e^ ± 0.09
		30	9.80 ^fg^ ± 0.09	9.80 ^ef^ ± 0.12	19.60 ^hi^ ± 0.10
	14	0	9.40 ^f^ ± 0.16	10.31 ^g^ ± 0.19	19.71 ^hi^ ± 0.18
		15	8.58 ^d^ ± 0.10	9.44 ^e^ ± 0.17	18.02 ^g^ ± 0.08
		30	9.72 ^f^ ± 0.15	10.76 ^gh^ ± 0.13	20.48 ^i^ ± 0.26
Clone BOR-17	1	0	7.93 ^c^ ± 0.30	7.69 ^b^ ± 0.18	15.62 ^c^ ± 0.15
		15	7.83 ^c^ ± 0.07	8.91 ^d^ ± 0.25	16.75 ^ef^ ± 0.18
		30	8.57 ^d^ ± 0.12	9.28 ^de^ ± 0.21	17.85 ^g^ ± 0.09
	7	0	6.54 ^a^ ± 0.20	7.20 ^a^ ± 0.20	13.74 ^a^ ± 0.21
		15	9.54 ^f^ ± 0.13	8.97 ^d^ ± 0.16	18.51 ^gh^ ± 0.12
		30	8.87 ^de^ ± 0.05	8.87 ^cd^ ± 0.05	17.74 ^fg^ ± 0.20
	14	0	8.39 ^d^ ± 0.08	7.84 ^b^ ± 0.30	16.24 ^d^ ± 0.10
		15	9.43 ^f^ ± 0.17	8.88 ^cd^ ± 0.09	18.31 ^g^ ± 0.09
		30	9.81 ^fg^ ± 0.18	8.45 ^c^ ± 0.15	18.26 ^g^ ± 0.11
Clone BOR-19	1	0	8.84 ^de^ ± 0.12	10.45 ^g^ ± 0.06	19.29 ^h^ ± 0.10
		15	8.40 ^d^ ± 0.15	9.16 ^d^ ± 0.18	17.56 ^f^ ± 0.15
		30	8.95 ^e^ ± 0.22	8.83 ^cd^ ± 0.11	17.77 ^fg^ ± 0.25
	7	0	7.92 ^c^ ± 0.08	9.22 ^d^ ± 0.18	17.15 ^f^ ± 0.23
		15	8.83 ^de^ ± 0.14	8.83 ^cd^ ± 0.22	17.66 ^fg^ ± 0.22
		30	11.14 ^i^ ± 0.24	8.95 ^d^ ± 0.23	20.09 ^i^ ± 0.11
	14	0	7.92 ^c^ ± 0.27	8.73 ^c^ ± 0.10	16.65 ^e^ ± 0.09
		15	9.69 ^f^ ± 0.16	10.81 ^gh^ ± 0.12	20.50 ^ij^ ± 0.16
		30	10.04 ^g^ ± 0.05	10.04 ^f^ ± 0.09	20.08 ^i^ ± 0.10
Clone BOR-20	1	0	7.74 ^c^ ± 0.17	8.77 ^c^ ± 0.14	16.51 ^e^ ± 0.06
		15	7.83 ^c^ ± 0.09	7.58 ^ab^ ± 0.22	15.41 ^bc^ ± 0.19
		30	8.41 ^d^ ± 0.12	8.35 ^c^ ± 0.10	16.77 ^ef^ ± 0.15
	7	0	8.01 ^c^ ± 0.17	7.71 ^b^ ± 0.08	15.72 ^c^ ± 0.21
		15	9.15 ^e^ ± 0.26	9.03 ^d^ ± 0.17	18.18 ^g^ ± 0.22
		30	9.65 ^f^ ± 0.11	9.29 ^de^ ± 0.12	18.94 ^h^ ± 0.13
	14	0	8.90 ^e^ ± 0.16	9.03 ^d^ ± 0.22	17.93 ^g^ ± 0.06
		15	8.54 ^d^ ± 0.08	8.17 ^c^ ± 0.13	16.71 ^e^ ± 0.11
		30	7.93 ^c^ ± 0.10	7.33 ^a^ ± 0.07	15.26 ^b^ ± 0.15
Clone BOR-21	1	0	8.71 ^d^ ± 0.13	8.09 ^bc^ ± 0.13	16.80 ^ef^ ± 0.14
		15	8.89 ^de^ ± 0.05	9.01 ^d^ ± 0.13	17.90 ^g^ ± 0.07
		30	7.55 ^b^ ± 0.09	8.85 ^cd^ ± 0.22	16.40 ^e^ ± 0.09
	7	0	9.57 ^f^ ± 0.18	9.14 ^d^ ± 0.28	18.70 ^gh^ ± 0.13
		15	8.92 ^e^ ± 0.13	8.63 ^c^ ± 0.11	17.55 ^f^ ± 0.18
		30	8.03 ^c^ ± 0.16	7.72 ^b^ ± 0.17	15.75 ^c^ ± 0.07
	14	0	10.28 ^g^ ± 0.23	9.78 ^ef^ ± 0.07	20.07 ^i^ ± 0.16
		15	9.14 ^e^ ± 0.27	8.58 ^c^ ± 0.14	17.72 ^fg^ ± 0.20
		30	9.47 ^f^ ± 0.17	9.74 ^e^ ± 0.18	19.21 ^h^ ± 0.10
“Bluecrop”	1	0	9.56 ^f^ ± 0.11	9.08 ^d^ ± 0.21	18.64 ^gh^ ± 0.15
		15	10.22 ^gh^ ± 0.14	10.22 ^f^ ± 0.08	20.44 ^i^ ± 0.22
		30	9.05 ^e^ ± 0.16	10.39 ^g^ ± 0.12	19.44 ^h^ ± 0.26
	7	0	8.84 ^de^ ± 0.08	10.42 ^g^ ± 0.15	19.26 ^h^ ± 0.12
		15	10.64 ^h^ ± 0.11	10.58 ^g^ ± 0.26	21.22 ^j^ ± 0.09
		30	9.34 ^ef^ ± 0.16	9.52 ^e^ ± 0.10	18.87 ^gh^ ± 0.11
	14	0	8.91 ^e^ ± 0.10	10.42 ^g^ ± 0.13	19.33 ^h^ ± 0.13
		15	10.71 ^h^ ± 0.17	10.53 ^g^ ± 0.20	21.24 ^j^ ± 0.10
		30	10.01 ^g^ ± 0.26	9.77 ^ef^ ± 0.27	19.78 ^hi^ ± 0.17
“Duke”	1	0	8.75 ^de^ ± 0.14	8.69 ^c^ ± 0.16	17.44 ^f^ ± 0.20
		15	7.68 ^b^ ± 0.08	8.87 ^cd^ ± 0.09	16.55 ^e^ ± 0.09
		30	10.24 ^g^ ± 0.13	10.30 ^g^ ± 0.18	20.54 ^ij^ ± 0.21
	7	0	9.23 ^e^ ± 0.17	10.98 ^h^ ± 0.12	20.21 ^i^ ± 0.17
		15	11.31 ^i^ ± 0.10	11.43 ^i^ ± 0.18	22.74 ^k^ ± 0.14
		30	9.46 ^f^ ± 0.18	9.33 ^e^ ± 0.14	18.79 ^gh^ ± 0.15
	14	0	9.46 ^f^ ± 0.25	10.82 ^gh^ ± 0.09	20.28 ^i^ ± 0.10
		15	9.51 ^f^ ± 0.08	8.71 ^c^ ± 0.06	18.22 ^g^ ± 0.21
		30	11.91 ^j^ ± 0.06	11.66 ^i^ ± 0.22	23.57 ^l^ ± 0.20
Average	$\overset{-}{x}\;1\;\pm$ SD	0	8.88 ^b^ ± 0.80	9.03 ^a^ ± 0.92	17.91 ^b^ ± 1.54
		15	8.57 ^a^ ± 0.88	9.09 ^a^ ± 0.76	17.66 ^a^ ± 1.51
		30	9.07 ^c^ ± 1.03	9.66 ^c^ ± 1.06	18.73 ^c^ ± 2.01
	$\overset{-}{x}\;7\;\pm$ SD	0	8.47 ^a^ ± 1.11	9.11 ^a^ ± 1.25	17.57 ^a^ ± 2.19
		15	9.35 ^d^ ± 1.21	9.43 ^c^ ± 1.01	18.77 ^c^ ± 2.11
		30	9.51 ^d^ ± 0.88	9.11 ^a^ ± 0.64	18.62 ^c^ ± 1.34
	$\overset{-}{x}\;14\;\pm$ SD	0	8.85 ^b^ ± 0.88	9.45 ^c^ ± 1.04	18.31 ^bc^ ± 1.75
		15	9.25 ^cd^ ± 0.77	9.28 ^b^ ± 0.94	18.53 ^c^ ± 1.54
		30	9.88 ^e^ ± 1.09	9.66 ^c^ ± 1.32	19.55 ^d^ ± 2.32

Data are expressed as mean values (*n* = 3) ± SD; SD—standard deviation. Mean values within columns with different letters are significantly different (*p* < 0.05) according to the Tukey test.

## Data Availability

Data can be made available by contacting the authors.

## Appendix A

**Table A1:** Individual phenolic compounds and their content depending on different treatments for fruits of the clone BOR-9.

Compound		Rt	λ_max_	(M-H) m/z		Clone BOR-9								
(mg·100 g^−1^ d.w.)		(min)	(nm)	MS	MS/MS									
Storage Time (Days)						1			7			14		
Ozone Exposure Time (min)						0	15	30	0	15	30	0	15	30
Anthocyanins														
1.	Delphinidin-3-O-glucoside	2.22	276, 522	465^+^	303	6.43 ^b^ ± 0.08	7.77 ^d^ ± 0.12	6.36 ^b^ ± 0.04	8.99 ^e^ ± 0.11	7.37 ^c^ ± 0.12	20.35 ^g^ ± 0.37	7.44 ^c^ ± 0.16	6.07 ^a^ ± 0.16	10.97 ^f^ ± 0.31
2.	Delphinidin-3-O-galactoside	2.35	276, 522	465^+^	303	3.61 ^a^ ± 0.04	4.78 ^c^ ± 0.29	3.58 ^a^ ± 0..18	5.59 ^d^ ± 0.41	4.10 ^b^ ± 0.10	10.12 ^f^ ± 0.22	4.79 ^c^ ± 0.11	4.14 ^b^ ± 0.11	5.99 ^e^ ± 0.26
3.	Cyanidin-3-O-glucoside	2.61	276, 519	449^+^	287	5.83 ^a^ ± 0.33	6.63 ^c^ ± 0.06	5.88 ^a^ ± 0.12	5.63 ^a^ ± 0.33	5.71 ^a^ ± 0.21	16.03 ^e^ ± 0.11	6.43 ^c^ ± 0.09	6.12 ^b^ ± 0.06	10.01 ^d^ ± 0.41
4.	Cyanidin-3-O-galactoside	2.74	279, 516	449^+^	287	0.95 ^b^ ± 0.12	0.93 ^b^ ± 0.15	0.89 ^b^ ± 0.00	0.95 ^b^ ± 0.01	0.47 ^a^ ± 0.18	1.84 ^e^ ± 0.15	1.09 ^bc^ ± 0.16	1.57 ^d^ ± 0.13	1.29 ^c^ ± 0.15
5.	Petunidin-3-O-glucoside	2.80	276, 524	479^+^	317	4.18 ^a^ ± 0.11	4.87 ± 0.28	4.25 ^a^ ± 0.25	5.56 ^b^ ± 0.08	4.21 ^a^ ± 0.11	10.67 ^d^ ± 0.33	4.82 ^b^ ± 0.11	4.75 ^b^ ± 0.22	5.93 ^c^ ± 0.08
6.	Petunidin-3-O-galactoside	2.93	276, 522	479^+^	317	4.55 ^b^ ± 0.17	5.55 ^c^ ± 0.21	4.72 ^b^ ± 0.21	6.21 ^e^ ± 0.16	4.15 ^a^ ± 0.17	9.59 ± 0.15	5.99 ^d^ ± 0.34	6.02 ^d^ ± 0.03	6.30 ^e^ ± 0.10
7.	Petunidin-3-O-arabinoside	3.13	277, 526	449^+^	317	2.75 ^c^ ± 0.21	2.60 ^bc^ ± 0.10	2.56 ^b^ ± 0.31	2.78 ^c^ ± 0.26	2.17 ^a^ ± 0.22	6.17 ^e^ ± 0.18	2.57 ^b^ ± 0.24	2.58 ^b^ ± 0.19	4.16 ^d^ ± 0.12
8.	Malvidin-3-O-glucoside	3.33	276, 524	493^+^	331	8.86 ^a^ ± 0.37	11.34 ^c^ ± 0.45	9.43 ^b^ ± 0.07	12.39 ^d^ ± 0.34	9.62 ^b^ ± 0.35	17.43 ^e^ ± 0.28	10.47 ^c^ ± 0.42	11.62 ^c^ ± 0.22	11.47 ^c^ ± 0.24
9.	Malvidin-3-O-galactoside	3.47	276, 526	493^+^	331	7.23 ^ab^ ± 0.41	9.94 ^e^ ± 0.18	7.48 ^b^ ± 0.16	10.29 ^f^ ± 0.33	7.02 ^a^ ± 0.17	11.46 ± 0.19	9.69 ^d^ ± 0.08	9.80 ^de^ ± 0.16	8.91 ^c^ ± 0.16
10.	Peonidin-3-O-glucoside	3.69	277, 527	463^+^	301	6.21 ^a^ ± 0.27	6.49 ^bc^ ± 0.17	6.64 ^c^ ± 0.13	6.67 ^c^ ± 0.08	6.35 ^ab^ ± 0.01	11.44 ^f^ ± 0.36	6.29 ^a^ ± 0.21	6.96 ^d^ ± 0.11	8.46 ^e^ ± 0.15
11.	Peonidin-3-O-galactoside	3.86	278, 527	463^+^	301	0.09 ^a^ ± 0.01	0.11 ^ab^ ± 0.02	0.24 ^b^ ± 0.09	0.08 ^a^ ± 0.01	0.22 ^b^ ± 0.00	0.21 ^b^ ± 0.01	0.10 ^a^ ± 0.00	0.17 ^b^ ± 0.07	0.08 ^a^ ± 0.00
12.	Cyanidin-3-O-(6′′-acetyl)-glucoside	4.05	271, 520	491^+^	449, 287	0.74 ^c^ ± 0.21	0.64 ^b^ ± 0.25	0.52 ^ab^ ± 0.13	0.46 ^a^ ± 0.20	0.37 ^a^ ± 0.11	0.70 ^bc^ ± 0.10	0.75 ^c^ ± 0.15	0.73 ^c^ ± 0.01	0.71 ^bc^ ± 0.14
13.	Petunidin-3-O-(6′′-acetyl)-glucoside	4.19	277, 527	521^+^	479, 317	0.73 ^b^ ± 0.11	1.11 ^c^ ± 0.09	1.21 ^c^ ± 0.22	1.11 ^c^ ± 0.11	0.47 ^a^ ± 0.22	1.13 ^c^ ± 0.13	1.42 ^d^ ± 0.11	1.14 ^c^ ± 0.14	0.79 ^b^ ± 0.11
14.	Cyanidin-3-O-(6′′-malonyl)glucoside	4.42	276, 529	535^+^	449, 287	0.58 ^b^ ± 0.09	0.77 ^c^ ± 0.11	1.05 ^d^ ± 0.31	0.60 ^b^ ± 0.00	0.38 ^a^ ± 0.02	1.36 ^e^ ± 0.10	0.83 ^c^ ± 0.31	1.17 ^d^ ± 0.17	0.47 ^ab^ ± 0.09
15.	Cyanidin-3-O-(6′′-malonyl)galactoside	4.76	277, 529	535^+^	449, 287	1.64 ^b^ ± 0.28	3.01 ^e^ ± 0.26	2.53 ^d^ ± 0.22	1.71 ^bc^ ± 0.12	0.82 ^a^ ± 0.11	1.91 ^c^ ± 0.16	3.38 ^f^ ± 0.09	2.88 ^de^ ± 0.07	1.55 ^b^ ± 0.11
Other Phenolics														
16.	Chlorogenic acid	2.68	288, 324	353^−^	191	16.96 ^f^ ± 0.37	16.69 ^e^ ± 0.55	15.54 ^c^ ± 0.21	13.72 ^a^ ± 0.38	14.78 ^b^ ± 0.35	17.43 ^g^ ± 0.31	16.22 ^d^ ± 0.25	16.94 ^f^ ± 0.46	16.66 ^e^ ± 0.31
17.	Myricetin 3-O-glucoside	3.66	253, 354	479^-^	317	0.23 ^a^ ± 0.07	0.32 ^ab^ ± 0.12	0.42 ^b^ ± 0.15	0.31 ^a^ ± 0.01	0.54 ^c^ ± 0.14	1.07 ^d^ ± 0.07	0.30 ^a^ ± 0.10	0.47 ^bc^ ± 0.13	0.61 ^c^ ± 0.14
18.	Quercetin 3-O-rutinoside	4.19	255, 354	609^-^	301	0.46 ^ab^ ± 0.21	0.48 ^b^ ± 0.01	0.40 ^a^ ± 0.10	0.32 ^a^ ± 0.09	0.39 ^a^ ± 0.05	0.48 ^b^ ± 0.11	0.86 ^c^ ± 0..13	0.61 ^b^ ± 0.04	0.52 ^b^ ± 0.03
19.	Quercetin 3-O-glucoside	4.28	255, 355	463^-^	301	1.35 ^a^ ± 0.18	2.00 ^d^ ± 0.10	1.58 ^bc^ ± 0.17	1.36 ^a^ ± 0.24	1.67 ^c^ ± 0.24	2.17 ^e^ ± 0.15	2.50 ^f^ ± 0.21	2.03 ^d^ ± 0.23	1.70 ^c^ ± 0.20
20.	Quercetin 3-O-galactoside	4.38	255, 355	463^-^	301	0.81 ^a^ ± 0.15	0.86 ^a^ ± 0.22	0.89 ^ab^ ± 0.28	0.88 ^a^ ± 0.26	0.97 ^b^ ± 0.29	1.21 ^c^ ± 0.31	1.68 ^d^ ± 0.27	1.28 ^c^ ± 0.30	1.20 ^c^ ± 0.11
21.	Quercetin 3-O-pentoside I	4.63	255, 355	433^-^	301	0.18 ^a^ ± 0.07	0.20 ^a^ ± 0.05	0.17 ^a^ ± 0.05	0.08 ^a^ ± 0.00	0.14 ^a^ ± 0.03	0.14 ^a^ ± 0.08	0.32 ^b^ ± 0.11	0.22 ^ab^ ± 0.02	0.19 ^a^ ± 0.07
22.	Quercetin 3-O-pentoside II	4.73	255, 355	433^-^	301	0.22 ^a^ ± 0.03	0.39 ^ab^ ± 0.09	0.29 ^a^ ± 0.01	0.26 ^a^ ± 0.04	0.31 ^a^ ± 0.10	0.37 ^a^ ± 0.16	0.43 ^b^ ± 0.16	0.37 ^a^ ± 0.14	0.29 ^a^ ± 0.11
23.	Quercetin 3-O-pentoside III	4.76	255, 355	433^-^	301	0.13 ^a^ ± 0.03	0.27 ^a^ ± 0.04	0.21 ^a^ ± 0.04	0.32 ^b^ ± 0.09	0.28 ^a^ ± 0.06	0.29 ^ab^ ± 0.08	0.36 ^b^ ± 0.12	0.41 ^b^ ± 0.11	0.30 ^b^ ± 0.05
24.	Isorhamnetin 3-O-rutinoside	4.84	255, 355	623^-^	301	0.30 ^b^ ± 0.10	0.33 ^b^ ± 0.12	0.20 ^a^ ± 0.00	0.23 ^ab^ ± 0.01	0.29 ^b^ ± 0.09	0.13 ^a^ ± 0.01	0.41 ^b^ ± 0.07	0.25 ^b^ ± 0.09	0.07 ^a^ ± 0.00
25.	Quercetin 3-O-rhamnoside	4.95	255, 355	447^-^	301	0.07 ^a^ ± 0.00	0.28 ^ab^ ± 0.11	0.36 ^b^ ± 0.11	0.42 ^b^ ± 0.15	0.39 ^b^ ± 0.10	0.63 ^c^ ± 0.21	0.44 ^bc^ ± 0.04	0.60 ^c^ ± 0.10	0.44 ^bc^ ± 0.03
26.	Quercetin 3-O-glucuronide	5.02	255, 354	477^-^	285	0.25 ^b^ ± 0.11	0.26 ^b^ ± 0.03	0.16 ^b^ ± 0.01	0.14 ^ab^ ± 0.00	0.29 ^b^ ± 0.07	0.20 ^b^ ± 0.04	0.24 ^b^ ± 0.11	0.19 ^b^ ± 0.06	0.03 ^a^ ± 0.00
27.	Quercetin 3-O-(6′′-acetylo)-glucoside	5.08	255, 335	505^-^	301	0.41 ^a^ ± 0.10	0.49 ^b^ ± 0.11	0.26 ^a^ ± 0.03	0.38 ^a^ ± 0.14	0.47 ^ab^ ± 0.13	0.28 ^a^ ± 0.08	0.64 ^b^ ± 0.25	0.58 ^b^ ± 0.17	0.33 ^a^ ± 0.01
	Total					75.75 ^a^ ± 0.46	89.11 ^c^ ± 0.92	77.82 ^a^ ± 0.34	87.47 ^b^ ± 0.67	73.96 ^a^ ± 0.21	144.80 ^e^ ± 0.38	90.47 ^c^ ± 0.65	89.70 ^c^ ± 0.78	99.42 ^d^ ± 0.28

Data are expressed as mean values (*n* = 3) ± SD; SD—standard deviation. Mean values within rows with different letters are significantly different (*p* < 0.05) according to the Tukey test. + and − in MS column means ionisation mode—positive (+) and negative (−).

**Table A2:** Individual phenolic compounds identified by UPLC-PDA-MS/MS for clone BOR-12.

Compound		Rt	λmax	(M-H) m/z		Clone BOR-12								
(mg·100 g^−1^ d.w.)		(min)	(nm)	MS	MS/MS									
Storage Time (Days)						1			7			14		
Ozone Exposure Time (min)						0	15	30	0	15	30	0	15	30
Anthocyanins														
1.	Delphinidin-3-O-glucoside	2.22	276, 522	465^+^	303	9.17 ^e^ ± 0.22	9.06 ^e^ ± 0.27	4.89 ^b^ ± 0.21	4.55 ^a^ ± 0.11	7.94 ^d^ ± 0.21	10.84 ^f^ ± 0.37	7.99 ^d^ ± 0.09	7.46 ^c^ ± 0.05	14.99 ^g^ ± 0.33
2.	Delphinidin-3-O-galactoside	2.35	276, 522	465^+^	303	4.58 ^c^ ± 0.14	4.55 ^c^ ± 0.28	3.11 ^b^ ± 0.02	2.03 ^a^ ± 0.00	4.94 ^d^ ± 0.17	5.51 ^e^ ± 0.31	4.90 ^d^ ± 0.17	5.95 ^f^ ± 0.21	7.14 ^g^ ± 0.18
3.	Cyanidin-3-O-glucoside	2.61	276, 519	449^+^	287	8.09 ^e^ ± 0.16	6.82 ^b^ ± 0.06	4.96 ^a^ ± 0.16	4.85 ^a^ ± 0.17	7.15 ^c^ ± 0.09	9.80 ^f^ ± 0.14	7.32 ^d^ ± 0.16	7.04 ^c^ ± 0.15	11.54 ^g^ ± 0.32
4.	Cyanidin-3-O-galactoside	2.74	279, 516	449^+^	287	0.90 ^b^ ± 0.10	0.75 ^a^ ± 0.01	1.09 ^b^ ± 0.18	0.99 ^b^ ± 0.09	0.86 ^ab^ ± 0.26	0.66 ^a^ ± 0.19	1.66 ^c^ ± 0.11	1.97 ^d^ ± 0.17	1.08 ^b^ ± 0.11
5.	Petunidin-3-O-glucoside	2.80	276, 524	479^+^	317	6.10 ^e^ ± 0.12	5.36 ^c^ ± 0.11	4.28 ^a^ ± 0.11	4.36 ^a^ ± 0.16	4.78 ^b^ ± 0.21	6.12 ^e^ ± 0.05	5.73 ^d^ ± 0.10	5.26 ^c^ ± 0.09	7.89 ^f^ ± 0.09
6.	Petunidin-3-O-galactoside	2.93	276, 522	479^+^	317	5.77 ^c^ ± 0.18	5.03 ^b^ ± 0.14	5.21 ^b^ ± 0.16	4.05 ^a^ ± 0.16	5.42 ^bc^ ± 0.32	5.79 ^c^ ± 0.02	6.70 ^d^ ± 0.00	7.65 ^e^ ± 0.16	7.42 ^e^ ± 0.25
7.	Petunidin-3-O-arabinoside	3.13	277, 526	449^+^	317	3.89 ^d^ ± 0.31	2.61 ^ab^ ± 0.19	2.47 ^a^ ± 0.16	2.89 ^bc^ ± 0.21	2.72 ^b^ ± 0.03	4.16 ^e^ ± 0.00	2.92 ^c^ ± 0.41	2.89 ^bc^ ± 0.21	4.33 ^f^ ± 0.21
8.	Malvidin-3-O-glucoside	3.33	276, 524	493^+^	331	13.86 ^d^ ± 0.18	11.13 ^a^ ± 0.36	11.10 ^a^ ± 0.02	13.16 ^c^ ± 0.26	11.20 ^a^ ± 0.16	14.89 ^f^ ± 0.35	14.47 ^e^ ± 0.34	12.04 ^b^ ± 0.32	13.36 ^c^ ± 0.17
9.	Malvidin-3-O-galactoside	3.47	276, 526	493^+^	331	9.47 ^d^ ± 0.11	7.74 ^a^ ± 0.21	9.52 ^de^ ± 0.13	7.98 ^b^ ± 0.17	8.63 ^c^ ± 0.17	9.74 ^e^ ± 0.11	10.62 ^f^ ± 0.29	11.83 ^g^ ± 0.07	9.33 ^d^ ± 0.06
10.	Peonidin-3-O-glucoside	3.69	277, 527	463^+^	301	9.35 ^f^ ± 0.21	6.19 ^a^ ± 0.18	7.82 ^c^ ± 0.12	7.79 ^c^ ± 0.15	7.18 ^b^ ± 0.21	10.66 ^g^ ± 0.11	8.00 ^d^ ± 0.10	7.18 ^b^ ± 0.28	8.28 ^e^ ± 0.06
11.	Peonidin-3-O-galactoside	3.86	278, 527	463^+^	301	0.14 ^a^ ± 0.05	0.19 ^a^ ± 0.06	0.15 ^a^ ± 0.03	0.14 ^a^ ± 0.21	0.13 ^a^ ± 0.04	0.10 ^a^ ± 0.00	0.45 ^b^ ± 0.03	0.14 ^a^ ± 0.01	0.15 ^a^ ± 0.01
12.	Cyanidin-3-O-(6′′-acetyl)-glucoside	4.05	271, 520	491^+^	449, 287	0.95 ^b^ ± 0.13	0.41 ^a^ ± 0.11	0.50 ^a^ ± 0.10	0.54 ^a^ ± 0.14	0.54 ^a^ ± 0.11	0.61 ^a^ ± 0.12	0.97 ^b^ ± 0.14	1.05 ^b^ ± 0.21	0.57 ^a^ ± 0.00
13.	Petunidin-3-O-(6′′-acetyl)-glucoside	4.19	277, 527	521^+^	479, 317	0.92 ^c^ ± 0.20	0.74 ^b^ ± 0.15	1.08 ^c^ ± 0.08	0.46 ^a^ ± 0.07	0.98 ^c^ ± 0.34	0.83 ^bc^ ± 0.22	1.45 ^d^ ± 0.17	1.60 ^d^ ± 0.10	0.90 ^c^ ± 0.19
14.	Cyanidin-3-O-(6′′-malonyl)glucoside	4.42	276, 529	535^+^	449, 287	0.93 ^ab^ ± 0.11	0.93 ab ± 0.25	1.10 ^b^ ± 0.10	0.76 ^a^ ± 0.16	0.85 ^a^ ± 0.15	0.91 ^a^ ± 0.03	2.06 ^c^ ± 0.19	0.81 ^a^ ± 0.14	0.84 ^a^ ± 0.10
15.	Cyanidin-3-O-(6′′-malonyl)galactoside	4.76	277, 529	535^+^	449, 287	1.73 ^a^ ± 0.16	1.93 ^a^ ± 0.06	2.30 ^b^ ± 0.20	1.69 ^a^ ± 0.11	1.82 ^a^ ± 0.14	1.78 ^a^ ± 0.14	3.46 ^c^ ± 0.24	3.32 ^c^ ± 0.22	1.58 ^a^ ± 0.04
Other Phenolics														
16.	Chlorogenic acid	2.68	288, 324	353^−^	191	17.13 ^e^ ± 0.34	14.17 ^a^ ± 0.37	16.51 ^d^ ± 0.31	16.79 ^d^ ± 0.78	16.39 ^d^ ± 0.36	16.46 ^d^ ± 0.31	15.59 ^b^ ± 0.25	16.61 ^d^ ± 0.27	15.97 ^c^ ± 0.05
17.	Myricetin 3-O-glucoside	3.66	253, 354	479^−^	317	0.41 ^ab^ ± 0.11	0.47 ^b^ ± 0.02	0.59 ^bc^ ± 0.06	0.19 ^a^ ± 0.08	0.25 ^a^ ± 0.05	0.71 ^c^ ± 0.10	0.28 ^a^ ± 0.03	0.43 ^b^ ± 0.16	0.86 ^c^ ± 0.12
18.	Quercetin 3-O-rutinoside	4.19	255, 354	609^−^	301	0.62 ^b^ ± 0.21	0.41 ^a^ ± 0.13	0.63 ^b^ ± 0.17	0.84 ^c^ ± 0.14	0.46 ^ab^ ± 0.15	0.24 ^a^ ± 0.04	0.55 ^b^ ± 0.12	0.67 ^b^ ± 0.12	0.65 ^b^ ± 0.16
19.	Quercetin 3-O-glucoside	4.28	255, 355	463^−^	301	2.19 ^cd^ ± 0.38	0.96 ^a^ ± 0.12	2.12 ^c^ ± 0.03	2.35 ^d^ ± 0.16	1.48 ^b^ ± 0.01	1.45 ^b^ ± 0.12	1.91 ^c^ ± 0.15	2.38 ^d^ ± 0.23	2.93 ^e^ ± 0.21
20.	Quercetin 3-O-galactoside	4.38	255, 355	463^−^	301	1.47 ^c^ ± 0.22	0.68 ^a^ ± 0.23	1.13 ^b^ ± 0.06	1.27 ^bc^ ± 0.14	0.81 ^a^ ± 0.11	0.81 ^a^ ± 0.15	0.93 ^ab^ ± 0.19	1.48 ^c^ ± 0.11	1.78 ^d^ ± 0.12
21.	Quercetin 3-O-pentoside I	4.63	255, 355	433^−^	301	0.24 ^a^ ± 0.08	0.09 ^a^ ± 0.02	0.16 ^a^ ± 0.01	0.29 ^a^ ± 0.06	0.18 ^a^ ± 0.00	0.10 ^a^ ± 0.19	0.21 ^a^ ± 0.04	0.25 ^a^ ± 0.04	0.11 ^a^ ± 0.01
22.	Quercetin 3-O-pentoside II	4.73	255, 355	433^−^	301	0.33 ^a^ ± 0.11	0.16 ^a^ ± 0.03	0.34 ^a^ ± 0.12	0.39 ^ab^ ± 0.08	0.26 ^a^ ± 0.04	0.24 ^a^ ± 0.06	0.29 ^a^ ± 0.06	0.39 ^ab^ ± 0.15	0.55 ^b^ ± 0.02
23.	Quercetin 3-O-pentoside III	4.76	255, 355	433^−^	301	0.14 ^a^ ± 0.06	0.18 ^a^ ± 0.14	0.36 ^ab^ ± 0.14	0.30 ^a^ ± 0.10	0.19 ^a^ ± 0.01	0.43 ^b^ ± 0.10	0.19 ^a^ ± 0.00	0.31 ^a^ ± 0.08	0.11 ^a^ ± 0.01
24.	Isorhamnetin 3-O-rutinoside	4.84	255, 355	623^−^	301	0.34 ^b^ ± 0.03	0.15 ^a^ ± 0.08	0.16 ^a^ ± 0.06	0.29 ^b^ ± 0.02	0.34 ^b^ ± 0.10	0.20 ^a^ ± 0.01	0.32 ^b^ ± 0.12	0.28 ^ab^ ± 0.04	0.04 ^a^ ± 0.00
25.	Quercetin 3-O-rhamnoside	4.95	255, 355	447^−^	301	0.08 ^a^ ± 0.00	0.26 ^a^ ± 0.02	0.53 ^b^ ± 0.21	0.63 ^b^ ± 0.11	0.16 ^a^ ± 0.08	0.79 ^c^ ± 0.25	0.14 ^a^ ± 0.06	0.50 ^b^ ± 0.10	0.57 ^b^ ± 0.16
26.	Quercetin 3-O-glucuronide	5.02	255, 354	477^−^	285	0.36 ^b^ ± 0.09	0.21 ^a^ ± 0.03	0.08 ^a^ ± 0.02	0.26 ^b^ ± 0.09	0.25 ^ab^ ± 0.02	0.07 ^a^ ± 0.00	0.21 ^a^ ± 0.02	0.18 ^a^ ± 0.01	0.13 ^a^ ± 0.02
27.	Quercetin 3-*O*-(6′′-acetylo)-glucoside	5.08	255, 335	505^−^	301	0.49 ^b^ ± 0.13	0.37 ^b^ ± 0.14	0.31 ^b^ ± 0.01	0.78 ^c^ ± 0.12	0.44 ^b^ ± 0.13	0.31 ^b^ ± 0.02	0.40 ^b^ ± 0.10	0.55 ^bc^ ± 0.02	0.03 ^a^ ± 0.00
	Total					99.66 ^d^ ± 0.74	81.55 ^ab^ ± 0.29	82.48 ^b^ ± 0.82	80.62 ^a^ ± 0.45	86.39 ^c^ ± 0.61	104.22 ^e^ ± 0.38	99.71 ^d^ ± 0.51	100.23 ^d^ ± 0.59	113.13 ^f^ ± 0.44

Data are expressed as mean values (*n* = 3) ± SD; SD—standard deviation. Mean values within rows with different letters are significantly different (*p* < 0.05) according to the Tukey test. + and − in MS column means ionisation mode—positive (+) and negative (−).

**Table A3:** Individual phenolic compounds identified by UPLC-PDA-MS/MS for clone BOR-17.

Compound		Rt	λ_max_	(M-H) m/z		Clone BOR-17								
(mg·100 g^−1^ d.w.)		(min)	(nm)	MS	MS/MS									
Storage Time (Days)						1			7			14		
Ozone Exposure Time (min)						0	15	30	0	15	30	0	15	30
Anthocyanins														
1.	Delphinidin-3-O-glucoside	2.22	276, 522	465^+^	303	12.89 ^h^ ± 0.09	10.41 ^g^ ± 0.27	7.66 ^d^ ± 0.14	7.08 ^c^ ± 0.11	9.55 ^e^ ± 0.25	18.55 ^i^ ± 0.33	4.87 ^b^ ± 0.11	4.39 ^a^ ± 0.21	9.64 ^f^ ± 0.22
2.	Delphinidin-3-O-galactoside	2.35	276, 522	465^+^	303	6.90 ^f^ ± 0.24	0.96 ^a^ ± 0.15	3.97 ^c^ ± 0.06	5.52 ^e^ ± 0.30	4.36 ^d^ ± 0.05	7.98 ^g^ ± 0.22	3.91 ^c^ ± 0.25	2.19 ^b^ ± 0.24	6.60 ^f^ ± 0.11
3.	Cyanidin-3-O-glucoside	2.61	276, 519	449^+^	287	10.02 ^g^ ± 0.34	4.06 ^a^ ± 0.07	6.43 ^d^ ± 0.12	7.02 ^e^ ± 0.08	7.70 ^f^ ± 0.00	13.69 ^h^ ± 0.15	5.20 ^c^ ± 0.16	4.59 ^b^ ± 0.05	7.69 ^f^ ± 0.14
4.	Cyanidin-3-O-galactoside	2.74	279, 516	449^+^	287	1.21 ^b^ ± 0.13	0.99 ^ab^ ± 0.09	1.13 ^b^ ± 0.01	2.36 ^d^ ± 0.13	0.79 ^a^ ± 0.13	0.94 ^a^ ± 0.21	1.64 ^c^ ± 0.24	0.77 ^a^ ± 0.02	1.22 ^b^ ± 0.23
5.	Petunidin-3-O-glucoside	2.80	276, 524	479^+^	317	7.35 ^e^ ± 0.15	3.28 ^a^ ± 0.13	5.04 ^c^ ± 0.24	5.00 ^c^ ± 0.02	6.49 ^d^ ± 0.16	9.75 ^f^ ± 0.34	3.45 ^ab^ ± 0.09	3.84 ^b^ ± 0.15	5.29 ^c^ ± 0.11
6.	Petunidin-3-O-galactoside	2.93	276, 522	479^+^	317	7.34 ^e^ ± 0.08	1.45 ^a^ ± 0.01	5.34 ^c^ ± 0.22	7.46 ^e^ ± 0.12	5.62 ^c^ ± 0.32	8.10 ^f^ ± 0.11	5.41 ^c^ ± 0.23	3.81 ^b^ ± 0.31	6.72 ^d^ ± 0.22
7.	Petunidin-3-O-arabinoside	3.13	277, 526	449^+^	317	4.17 ^e^ ± 0.15	1.59 ^a^ ± 0.18	2.60 ^c^ ± 0.31	2.85 ^c^ ± 0.07	3.27 ^d^ ± 0.24	5.17 ^f^ ± 0.15	2.22 ^b^ ± 0.05	2.17 ^b^ ± 0.22	3.13 ^d^ ± 0.17
8.	Malvidin-3-O-glucoside	3.33	276, 524	493^+^	331	16.09 ^g^ ± 0.16	13.44 ^e^ ± 0.36	10.99 ^b^ ± 0.21	12.76 ^d^ ± 0.26	14.35 ^f^ ± 0.32	16.36 ^h^ ± 0.24	10.07 ^a^ ± 0.11	10.65 ^b^ ± 0.12	11.49 ^c^ ± 0.01
9.	Malvidin-3-O-galactoside	3.47	276, 526	493^+^	331	11.64 ^g^ ± 0.29	2.08 ^a^ ± 0.07	9.10 ^c^ ± 0.21	11.35 ^f^ ± 0.33	9.30 ^cd^ ± 0.13	10.86 ^e^ ± 0.32	9.68 ^d^ ± 0.10	6.77 ^b^ ± 0.01	10.87 ^e^ ± 0.11
10.	Peonidin-3-O-glucoside	3.69	277, 527	463^+^	301	9.83 ^h^ ± 0.18	3.95 ^a^ ± 0.12	6.13 ^b^ ± 0.03	7.73 ^e^ ± 0.14	8.59 ^f^ ± 0.00	8.98 ^g^ ± 0.21	6.82 ^c^ ± 0.25	7.44 ^d^ ± 0.12	7.74 ^e^ ± 0.08
11.	Peonidin-3-O-galactoside	3.86	278, 527	463^+^	301	0.10 ^a^ ± 0.03	0.24 ^a^ ± 0.15	0.13 ^a^ ± 0.00	0.26 ^a^ ± 0.18	0.14 ^a^ ± 0.02	0.10 ^a^ ± 0.02	0.14 ^a^ ± 0.01	0.29 ^a^ ± 0.09	0.14 ^a^ ± 0.03
12.	Cyanidin-3-O-(6′′-acetyl)-glucoside	4.05	271, 520	491^+^	449, 287	0.95 ^c^ ± 0.13	0.12 ^a^ ± 0.06	0.55 ^b^ ± 0.11	1.04 ^cd^ ± 0.09	0.40 ^b^ ± 0.10	0.75 ^bc^ ± 0.15	0.94 ^c^ ± 0.22	0.50 ^b^ ± 0.00	1.21 ^d^ ± 0.04
13.	Petunidin-3-O-(6′′-acetyl)-glucoside	4.19	277, 527	521^+^	479, 317	0.93 ^c^ ± 0.11	0.24 ^a^ ± 0.05	0.91 ^c^ ± 0.21	1.57 ^d^ ± 0.12	0.45 ^ab^ ± 0.12	0.67 ^b^ ± 0.09	1.33 ^d^ ± 0.12	0.74 ^bc^ ± 0.21	1.45 ^d^ ± 0.21
14.	Cyanidin-3-O-(6′′-malonyl)glucoside	4.42	276, 529	535^+^	449, 287	0.75 ^a^ ± 0.07	1.09 ^bc^ ± 0.03	0.74 ^a^ ± 0.04	1.28 ^c^ ± 0.11	1.02 ^b^ ± 0.24	0.53 ^a^ ± 0.15	0.90 ^b^ ± 0.09	1.64 ^d^ ± 0.28	0.76 ^ab^ ± 0.17
15.	Cyanidin-3-O-(6′′-malonyl)galactoside	4.76	277, 529	535^+^	449, 287	1.83 ^d^ ± 0.21	0.69 ^a^ ± 0.11	2.35 ^e^ ± 0.31	3.33 ^f^ ± 0.23	1.41 ^c^ ± 0.11	1.05 ^b^ ± 0.11	3.16 ^f^ ± 0.17	2.31 ^e^ ± 0.32	3.15 ^f^ ± 0.13
Other Phenolics														
16.	Chlorogenic acid	2.68	288, 324	353^−^	191	15.12 ^a^ ± 0.35	15.12 ^a^ ± 0.37	15.78 ^b^ ± 0.37	16.32 ^c^ ± 0.16	17.35 ^d^ ± 0.33	17.70 ^e^ ± 0.28	16.29 ^c^ ± 0.32	15.28 ^a^ ± 0.09	15.86 ^b^ ± 0.31
17.	Myricetin 3-O-glucoside	3.66	253, 354	479^−^	317	0.61 ^b^ ± 0.17	0.26 ^a^ ± 0.13	0.37 ^a^ ± 0.09	0.23 ^a^ ± 0.03	0.44 ^a^ ± 0.16	0.62 ^b^ ± 0.11	0.54 ^ab^ ± 0.09	0.56 ^b^ ± 0.12	0.62 ^b^ ± 0.15
18.	Quercetin 3-O-rutinoside	4.19	255, 354	609^−^	301	0.57 ^b^ ± 0.13	0.28 ^a^ ± 0.09	0.45 ^a^ ± 0.04	0.79 ^c^ ± 0.11	0.89 ^c^ ± 0.25	0.49 ^b^ ± 0.22	0.60 ^b^ ± 0.10	0.47 ^ab^ ± 0.09	0.52 ^b^ ± 0.06
19.	Quercetin 3-O-glucoside	4.28	255, 355	463^−^	301	1.93 ^bc^ ± 0.11	0.85 ^a^ ± 0.15	1.72 ^b^ ± 0.21	2.95 ^d^ ± 0.27	2.95 ^d^ ± 0.31	1.69 ^b^ ± 0.24	1.90 ^b^ ± 0.23	2.01 ^c^ ± 0.22	2.28 ^c^ ± 0.32
20.	Quercetin 3-O-galactoside	4.38	255, 355	463^−^	301	0.82 ^b^ ± 0.18	0.61 ^a^ ± 0.18	0.86 ^b^ ± 0.12	1.35 ^cd^ ± 0.17	1.49 ^d^ ± 0.22	1.24 ^c^ ± 0.19	1.12 ^c^ ± 0.28	1.14 ^c^ ± 0.13	1.12 ^c^ ± 0.18
21.	Quercetin 3-O-pentoside I	4.63	255, 355	433^−^	301	0.13 ^a^ ± 0.07	0.09 ^a^ ± 0.01	0.20 ^a^ ± 0.10	0.27 ^a^ ± 0.02	0.30 ^a^ ± 0.10	0.18 ^a^ ± 0.06	0.19 ^a^ ± 0.03	0.16 ^a^ ± 0.05	0.16 ^a^ ± 0.09
22.	Quercetin 3-O-pentoside II	4.73	255, 355	433^−^	301	0.31 ^a^ ± 0.10	0.15 ^a^ ± 0.00	0.27 ^a^ ± 0.09	0.49 ^bc^ ± 0.11	0.54 ^c^ ± 0.12	0.33 ^ab^ ± 0.12	0.34 ^b^ ± 0.06	0.36 ^b^ ± 0.11	0.37 ^b^ ± 0.07
23.	Quercetin 3-O-pentoside III	4.76	255, 355	433^−^	301	0.32 ^a^ ± 0.06	0.14 ^a^ ± 0.03	0.15 ^a^ ± 0.05	0.20 ^a^ ± 0.02	0.41 ^b^ ± 0.07	0.37 ^b^ ± 0.11	0.38 ^b^ ± 0.11	0.28 ^a^ ± 0.08	0.35 ^ab^ ± 0.11
24.	Isorhamnetin 3-O-rutinoside	4.84	255, 355	623^−^	301	0.30 ^ab^ ± 0.08	0.14 ^a^ ± 0.04	0.29 ± 0.03	0.27 ^a^ ± 0.03	0.48 ^b^ ± 0.13	0.10 ^a^ ± 0.00	0.34 ^b^ ± 0.01	0.21 ^a^ ± 0.02	0.14 ^a^ ± 0.08
25.	Quercetin 3-O-rhamnoside	4.95	255, 355	447^−^	301	0.39 ^a^ ± 0.11	0.21 ^a^ ± 0.00	0.17 ^a^ ± 0.07	0.26 ^a^ ± 0.10	0.38 ^a^ ± 0.21	0.62 ^b^ ± 0.07	0.61 ^b^ ± 0.15	0.42 ^ab^ ± 0.12	0.60 ^b^ ± 0.10
26.	Quercetin 3-O-glucuronide	5.02	255, 354	477^−^	285	0.24 ^a^ ± 0.02	0.05 ^a^ ± 0.01	0.22 ^a^ ± 0.02	0.29 ^a^ ± 0.09	0.38 ^a^ ± 0.09	0.12 ^a^ ± 0.04	0.25 ^a^ ± 0.05	0.10 ^a^ ± 0.01	0.18 ^a^ ± 0.03
27.	Quercetin 3-O-(6′′-acetylo)-glucoside	5.08	255, 335	505^−^	301	0.48 ^ab^ ± 0.02	0.21 ^a^ ± 0.08	0.25 ^a^ ± 0.11	0.73 ^b^ ± 0.22	0.84 ^b^ ± 0.17	0.47 ^a^ ± 0.03	0.65 ^b^ ± 0.01	0.35 ^a^ ± 0.09	0.21 ^a^ ± 0.03
	Total					113.21 ^f^ ± 0.33	62.69 ^a^ ± 0.78	83.81 ^c^ ± 0.66	100.77 ^c^ ± 0.39	99.90 ^c^ ± 0.48	127.39 ^e^ ± 0.56	82.96 ^c^ ± 0.51	73.41 ^b^ ± 0.69	99.51 ^c^ ± 0.74

Data are expressed as mean values (*n* = 3) ± SD; SD—standard deviation. Mean values within rows with different letters are significantly different (*p* < 0.05) according to the Tukey test. + and − in MS column means ionisation mode—positive (+) and negative (−).

**Table A4:** Individual phenolic compounds identified by UPLC-PDA-MS/MS for clone BOR-19.

Compound		Rt	λ_max_	(M-H) m/z		Clone BOR-19								
(mg·100 g^−1^ d.w.)		(min)	(nm)	MS	MS/MS									
Storage Time (Days)						1			7			14		
Ozone Exposure Time (min)						0	15	30	0	15	30	0	15	30
Anthocyanins														
1.	Delphinidin-3-O-glucoside	2.22	276, 522	465^+^	303	10.30 ^d^ ± 0.22	3.93 ^a^ ± 0.11	12.03 ^f^ ± 0.22	3.86 ^a^ ± 0.09	7.79 ^c^ ± 0.21	15.53 ^h^ ± 0.27	11.43 ^e^ ± 0.21	6.12 ^b^ ± 0.11	14.24 ^g^ ± 0.11
2.	Delphinidin-3-O-galactoside	2.35	276, 522	465^+^	303	6.30 ^ef^ ± 0.11	6.74 ^f^ ± 0.16	4.51 ^c^ ± 0.13	1.95 ^a^ ± 0.11	6.00 ^de^ ± 0.10	7.75 ^g^ ± 0.11	6.17 ^e^ ± 0.09	2.91 ^b^ ± 0.15	5.87 ^d^ ± 0.15
3.	Cyanidin-3-O-glucoside	2.61	276, 519	449^+^	287	8.78 ^e^ ± 0.21	1.08 ^a^ ± 0.07	9.65 ^g^ ± 0.08	3.99 ^b^ ± 0.15	6.49 ^d^ ± 0.17	11.92 ^h^ ± 0.21	9.15 ^f^ ± 0.11	5.97 ^c^ ± 0.17	11.53 ^h^ ± 0.08
4.	Cyanidin-3-O-galactoside	2.74	279, 516	449^+^	287	1.40 ^c^ ± 0.17	5.53 ^d^ ± 0.23	0.60 ^a^ ± 0.10	0.47 ^a^ ± 0.21	1.41 ^c^ ± 0.11	0.93 ^b^ ± 0.09	0.66 ^a^ ± 0.21	0.63 ^a^ ± 0.09	0.73 ^ab^ ± 0.09
5.	Petunidin-3-O-glucoside	2.80	276, 524	479^+^	317	5.80 ^d^ ± 0.11	5.33 ^c^ ± 0.11	7.36 ^f^ ± 0.31	3.56 ^a^ ± 0.21	4.78 ^b^ ± 0.21	7.49 ^f^ ± 0.21	6.22 ^e^ ± 0.02	4.90 ^b^ ± 0.10	7.98 ^g^ ± 0.13
6.	Petunidin-3-O-galactoside	2.93	276, 522	479^+^	317	7.14 ^f^ ± 0.21	2.79 ^a^ ± 0.11	5.44 ^d^ ± 0.21	3.32 ^b^ ± 0.06	6.67 ^e^ ± 0.06	7.40 ^f^ ± 0.10	6.27 ^e^ ± 0.06	4.28 ^c^ ± 0.12	6.21 ^e^ ± 0.17
7.	Petunidin-3-O-arabinoside	3.13	277, 526	449^+^	317	3.54 ^c^ ± 0.09	10.49 ^e^ ± 0.27	3.64 ^c^ ± 0.09	2.37 ^a^ ± 0.11	2.41 ^a^ ± 0.21	3.74 ^c^ ± 0.09	3.31 ^bc^ ± 0.00	3.05 ^b^ ± 0.07	4.98 ^d^ ± 0.08
8.	Malvidin-3-O-glucoside	3.33	276, 524	493^+^	331	11.83 ^c^ ± 0.22	8.23 ^a^ ± 0.09	14.14 ^f^ ± 0.25	12.83 ^e^ ± 0.17	10.24 ^b^ ± 0.18	12.98 ^e^ ± 0.14	12.39 ^d^ ± 0.21	12.66 ^de^ ± 0.22	14.48 ^f^ ± 0.21
9.	Malvidin-3-O-galactoside	3.47	276, 526	493^+^	331	10.60 ^f^ ± 0.29	5.92 ^a^ ± 0.16	7.19 ^b^ ± 0.24	8.19 ^c^ ± 0.31	10.63 ^f^ ± 0.28	9.46 ^e^ ± 0.18	9.20 ^e^ ± 0.10	8.31 ^c^ ± 0.18	8.89 ^d^ ± 0.04
10.	Peonidin-3-O-glucoside	3.69	277, 527	463^+^	301	7.91 ^d^ ± 0.17	0.11 ^a^ ± 0.05	7.72 ^d^ ± 0.07	10.95 ^g^ ± 0.22	5.28 ^b^ ± 0.29	7.46 ^cd^ ± 0.22	7.26 ^c^ ± 0.31	9.26 ^e^ ± 0.16	9.81 ^f^ ± 0.31
11.	Peonidin-3-O-galactoside	3.86	278, 527	463^+^	301	0.15 ^a^ ± 0.09	0.37 ^b^ ± 0.09	0.41 ^b^ ± 0.09	0.10 ^a^ ± 0.00	0.14 ^a^ ± 0.09	0.09 ^a^ ± 0.00	0.11 ^a^ ± 0.02	0.07 ^a^ ± 0.00	0.09 ^a^ ± 0.04
12.	Cyanidin-3-O-(6′′-acetyl)-glucoside	4.05	271, 520	491^+^	449, 287	1.11 ^c^ ± 0.03	0.79 ^b^ ± 0.11	0.40 ^a^ ± 0.03	0.58 ^a^ ± 0.12	0.81 ^b^ ± 0.11	0.43 ^a^ ± 0.04	0.61 ^ab^ ± 0.21	0.37 ^a^ ± 0.13	0.35 ^a^ ± 0.08
13.	Petunidin-3-O-(6′′-acetyl)-glucoside	4.19	277, 527	521^+^	479, 317	1.35 ^d^ ± 0.04	0.70 ^b^ ± 0.10	1.09 ^c^ ± 0.05	0.65 ^b^ ± 0.06	1.45 ^d^ ± 0.24	0.56 ^ab^ ± 0.13	0.87 ^b^ ± 0.06	0.32 ^a^ ± 0.08	0.32 ^a^ ± 0.11
14.	Cyanidin-3-O-(6′′-malonyl)glucoside	4.42	276, 529	535^+^	449, 287	0.91 ^ab^ ± 0.11	1.53 ^c^ ± 0.16	2.00 ^d^ ± 0.31	1.10 ^b^ ± 0.10	0.78 ^a^ ± 0.15	0.59 ^a^ ± 0.11	0.63 ^a^ ± 0.04	0.69 ^a^ ± 0.03	0.54 ^a^ ± 0.1
15.	Cyanidin-3-O-(6′′-malonyl)galactoside	4.76	277, 529	535^+^	449, 287	2.78 ^e^ ± 0.21	2.35 ^d^ ± 0.26	2.14 ^cd^ ± 0.26	1.96 ^c^ ± 0.25	3.08 ^f^ ± 0.16	1.20 ^b^ ± 0.16	1.32 ^b^ ± 0.16	1.28 ^b^ ± 0.11	0.89 ^a^ ± 0.05
Other Phenolics														
16.	Chlorogenic acid	2.68	288, 324	353^−^	191	16.00 ^b^ ± 0.34	13.47 ^a^ ± 0.22	15.75 ^b^ ± 0.31	16.24 ^c^ ± 0.36	15.98 ^b^ ± 0.31	16.43 ^c^ ± 0.21	15.88 ^b^ ± 0.12	16.33 ^c^ ± 0.22	17.86 ^d^ ± 0.33
17.	Myricetin 3-O-glucoside	3.66	253, 354	479^−^	317	0.43 ^a^ ± 0.21	0.32 ^a^ ± 0.02	0.59 ^b^ ± 0.05	0.28 ^a^ ± 0.09	0.24 ^a^ ± 0.04	0.54 ^ab^ ± 0.09	0.25 ^a^ ± 0.01	0.29 ^a^ ± 0.09	0.77 ^b^ ± 0.06
18.	Quercetin 3-O-rutinoside	4.19	255, 354	609^−^	301	0.71 ^ab^ ± 0.18	0.51 ^a^ ± 0.03	0.47 ^a^ ± 0.07	0.46 ^a^ ± 0.11	0.73 ^b^ ± 0.15	0.47 ^a^ ± 0.17	0.77 ^b^ ± 0.09	0.81 ^b^ ± 0.22	0.60 ^a^ ± 0.10
19.	Quercetin 3-O-glucoside	4.28	255, 355	463^−^	301	1.49 ^a^ ± 0.24	1.95 ^b^ ± 0.22	2.23 ^c^ ± 0.25	1.65 ^a^ ± 0.15	2.23 ^c^ ± 0.07	2.17 ^bc^ ± 0.26	1.97 ^b^ ± 0.25	2.20 ^c^ ± 0.28	1.94 ^b^ ± 0.07
20.	Quercetin 3-O-galactoside	4.38	255, 355	463^−^	301	0.97 ^ab^ ± 0.08	1.13 ^b^ ± 0.09	1.08 ^b^ ± 0.16	0.83 ^c^ ± 0.18	1.52 ^c^ ± 0.22	1.23 ^bc^ ± 0.22	1.51 ^c^ ± 0.31	1.65 ^c^ ± 0.19	1.47 ^c^ ± 0.21
21.	Quercetin 3-O-pentoside I	4.63	255, 355	433^−^	301	0.21 ^a^ ± 0.09	0.16 ^a^ ± 0.00	0.19 ^a^ ± 0.01	0.18 ^a^ ± 0.06	0.26 ^a^ ± 0.09	0.18 ^a^ ± 0.09	0.16 ^a^ ± 0.00	0.29 ^a^ ± 0.09	0.19 ^a^ ± 0.07
22.	Quercetin 3-O-pentoside II	4.73	255, 355	433^−^	301	0.28 ^a^ ± 0.01	0.32 ^a^ ± 0.10	0.35 ^a^ ± 0.04	0.31 ^a^ ± 0.09	0.35 ^a^ ± 0.11	0.34 ^a^ ± 0.01	0.34 ^a^ ± 0.11	0.38 ^a^ ± 0.12	0.30 ^a^ ± 0.09
23.	Quercetin 3-O-pentoside III	4.76	255, 355	433^−^	301	0.34 ^a^ ± 0.16	0.23 ^a^ ± 0.09	0.21 ^a^ ± 0.07	0.26 ^a^ ± 0.09	0.19 ^a^ ± 0.06	0.26 ^a^ ± 0.07	0.23 ^a^ ± 0.06	0.22 ^a^ ± 0.01	0.18 ^a^ ± 0.00
24.	Isorhamnetin 3-O-rutinoside	4.84	255, 355	623^−^	301	0.37 ^b^ ± 0.21	0.28 ^ab^ ± 0.02	0.18 ^a^ ± 0.09	0.39 ^b^ ± 0.11	0.36 ^b^ ± 0.07	0.15 ^a^ ± 0.00	0.35 ^b^ ± 0.01	0.40 ^b^ ± 0.10	0.12 ^a^ ± 0.03
25.	Quercetin 3-O-rhamnoside	4.95	255, 355	447^−^	301	0.41 ^b^ ± 0.09	0.27 ^a^ ± 0.06	0.31 ^ab^ ± 0.09	0.25 ^a^ ± 0.05	0.22 ^a^ ± 0.03	0.56 ^b^ ± 0.15	0.44 ^b^ ± 0.03	0.16 ^a^ ± 0.02	0.27 ^a^ ± 0.04
26.	Quercetin 3-O-glucuronide	5.02	255, 354	477^−^	285	0.24 ^a^ ± 0.07	0.16 ^a^ ± 0.04	0.27 ^a^ ± 0.01	0.26 ^a^ ± 0.09	0.25 ^a^ ± 0.05	0.12 ^a^ ± 0.01	0.24 ^a^ ± 0.08	0.29 ^a^ ± 0.08	0.05 ^a^ ± 0.00
27.	Quercetin 3-O-(6′′-acetylo)-glucoside	5.08	255, 335	505^−^	301	0.61 ^b^ ± 0.01	0.49 ^ab^ ± 0.07	0.24 ^a^ ± 0.03	0.49 ^ab^ ± 0.12	0.62 ^b^ ± 0.13	0.43 ^a^ ± 0.22	0.77 ^b^ ± 0.11	0.67 ^b^ ± 0.14	0.29 ^a^ ± 0.09
	Total					101.97 ^g^ ± 0.49	75.17 ^a^ ± 0.71	100.19 ^f^ ± 0.39	77.50 ^b^ ± 0.79	90.91 ^d^ ± 0.29	110.39 ^h^ ± 0.61	98.51 ^e^ ± 0.41	84.52 ^c^ ± 0.59	110.94 ^h^ ± 0.49

Data are expressed as mean values (*n* = 3) ± SD; SD—standard deviation. Mean values within rows with different letters are significantly different (*p* < 0.05) according to the Tukey test. + and − in MS column means ionisation mode—positive (+) and negative (−).

**Table A5:** Individual phenolic compounds identified by UPLC-PDA-MS/MS for clone BOR-20.

Compound		Rt	λ_max_	(M-H) m/z		Clone BOR-20								
(mg·100 g^−1^ d.w.)		(min)	(nm)	MS	MS/MS									
Storage Time (Days)						1			7			14		
Ozone Exposure Time (min)						0	15	30	0	15	30	0	15	30
Anthocyanins														
1.	Delphinidin-3-O-glucoside	2.22	276, 522	465^+^	303	7.84 ^c^ ± 0.22	7.14 ^b^ ± 0.13	7.90 ^c^ ± 0.10	4.26 ^a^ ± 0.17	8.26 ^d^ ± 0.06	11.37 ^g^ ± 0.31	9.22 ^e^ ± 0.13	14.20 ^h^ ± 0.21	10.58 ^f^ ± 0.09
2.	Delphinidin-3-O-galactoside	2.35	276, 522	465^+^	303	5.08 ^e^ ± 0.13	3.17 ^b^ ± 0.14	4.80 ^d^ ± 0.12	2.24 ^a^ ± 0.13	4.21 ^c^ ± 0.11	7.23 ^i^ ± 0.19	5.69 ^g^ ± 0.04	6.13 ^h^ ± 0.09	5.44 ^f^ ± 0.11
3.	Cyanidin-3-O-glucoside	2.61	276, 519	449^+^	287	6.39 ^b^ ± 0.18	6.28 ^b^ ± 0.09	7.01 ^c^ ± 0.31	4.55 ^a^ ± 0.05	6.67 ^b^ ± 0.05	8.06 ^d^ ± 0.09	8.20 ^d^ ± 0.15	9.43 ^f^ ± 0.15	9.03 ^e^ ± 0.16
4.	Cyanidin-3-O-galactoside	2.74	279, 516	449^+^	287	1.03 ^c^ ± 0.06	0.75 ^ab^ ± 0.11	1.42 ^d^ ± 0.09	0.79 ^b^ ± 0.23	0.56 ^a^ ± 0.09	1.15 ^c^ ± 0.01	1.32 ^cd^ ± 0.12	1.15 ^c^ ± 0.11	0.68 ^a^ ± 0.08
5.	Petunidin-3-O-glucoside	2.80	276, 524	479^+^	317	4.14 ^b^ ± 0.18	5.21 ^d^ ± 0.18	5.40 ^de^ ± 0.08	3.78 ^a^ ± 0.13	4.99 ^c^ ± 0.11	5.79 ^e^ ± 0.14	5.99 ^e^ ± 0.22	7.59 ^f^ ± 0.22	5.68 ^e^ ± 0.21
6.	Petunidin-3-O-galactoside	2.93	276, 522	479^+^	317	5.09 ^c^ ± 0.19	4.53 ^b^ ± 0.21	6.24 ^e^ ± 0.15	4.05 ^a^ ± 0.04	4.58 ^b^ ± 0.18	6.99 ^g^ ± 0.23	7.05 ^h^ ± 0.18	6.64 ^f^ ± 0.13	5.63 ^d^ ± 0.25
7.	Petunidin-3-O-arabinoside	3.13	277, 526	449^+^	317	2.62 ^b^ ± 0.11	2.87 ^b^ ± 0.15	2.72 ^b^ ± 0.06	2.22 ^a^ ± 0.21	2.72 ^b^ ± 0.24	3.36 ^c^ ± 0.09	3.23 ^c^ ± 0.11	3.47 ^cd^ ± 0.15	3.76 ^d^ ± 0.17
8.	Malvidin-3-O-glucoside	3.33	276, 524	493^+^	331	9.34 ^a^ ± 0.25	11.15 ^d^ ± 0.09	11.63 ^e^ ± 0.26	10.38 ^b^ ± 0.33	10.95 ^c^ ± 0.38	11.96 ^f^ ± 0.23	12.66 ^g^ ± 0.32	15.27 ^h^ ± 0.09	11.60 ^e^ ± 0.10
9.	Malvidin-3-O-galactoside	3.47	276, 526	493^+^	331	8.75 ^d^ ± 0.09	7.47 ^a^ ± 0.11	9.34 ^e^ ± 0.13	7.75 ^b^ ± 0.08	7.46 ^a^ ± 0.04	10.51 ^g^ ± 0.31	10.23 ^g^ ± 0.14	9.93 ^f^ ± 0.06	8.47 ^c^ ± 0.14
10.	Peonidin-3-O-glucoside	3.69	277, 527	463^+^	301	6.57 ^b^ ± 0.14	7.16 ^c^ ± 0.16	6.23 ^a^ ± 0.16	7.70 ^d^ ± 0.21	7.52 ^d^ ± 0.13	7.80 ^d^ ± 0.12	8.12 ^e^ ± 0.19	7.01 ^c^ ± 0.13	9.04 ^f^ ± 0.09
11.	Peonidin-3-O-galactoside	3.86	278, 527	463^+^	301	0.07 ^a^ ± 0.03	0.09 ^a^ ± 0.01	0.16 ^a^ ± 0.07	0.10 ^a^ ± 0.00	0.06 ^a^ ± 0.00	0.14 ^a^ ± 0.05	0.15 ^a^ ± 0.02	0.04 ^a^ ± 0.01	0.20 ^a^ ± 0.02
12.	Cyanidin-3-O-(6′′-acetyl)-glucoside	4.05	271, 520	491^+^	449, 287	0.85 ^c^ ± 0.11	0.35 ^a^ ± 0.05	0.77 ^bc^ ± 0.11	0.52 ^ab^ ± 0.11	0.31 ^a^ ± 0.11	0.81 ^c^ ± 0.09	0.62 ^b^ ± 0.11	0.66 ^b^ ± 0.11	0.83 ^c^ ± 0.12
13.	Petunidin-3-O-(6′′-acetyl)-glucoside	4.19	277, 527	521^+^	479, 317	0.99 ^bc^ ± 0.14	0.32 ^a^ ± 0.08	1.09 ^c^ ± 0.06	0.72 ^b^ ± 0.16	0.30 ^a^ ± 0.09	1.60 ^d^ ± 0.13	1.19 ^c^ ± 0.21	0.36 ^a^ ± 0.06	1.06 ^c^ ± 0.16
14.	Cyanidin-3-O-(6′′-malonyl)glucoside	4.42	276, 529	535^+^	449, 287	0.59 ^b^ ± 0.21	0.50 ^ab^ ± 0.10	0.85 ^bc^ ± 0.15	0.91 ^c^ ± 0.05	0.32 ^a^ ± 0.07	0.77 ^b^ ± 0.14	0.96 ^c^ ± 0.08	0.22 ^a^ ± 0.05	0.99 ^c^ ± 0.04
15.	Cyanidin-3-O-(6′′-malonyl)galactoside	4.76	277, 529	535^+^	449, 287	2.58 ^e^ ± 0.29	0.89 ^b^ ± 0.09	2.20 ^d^ ± 0.17	1.72 ^c^ ± 0.11	0.71 ^ab^ ± 0.15	3.12 ^f^ ± 0.09	2.07 ^d^ ± 0.18	0.51 ^a^ ± 0.12	1.80 ^c^ ± 0.15
Other Phenolics														
16.	Chlorogenic acid	2.68	288, 324	353^−^	191	14.75 ^a^ ± 0.29	15.52 ^bc^ ± 0.22	15.67 ^c^ ± 0.21	15.35 ^b^ ± 0.15	15.88 ^c^ ± 0.36	18.63 ^f^ ± 0.09	17.07 ^d^ ± 0.33	16.89 ^d^ ± 0.35	17.57 ^e^ ± 0.25
17.	Myricetin 3-O-glucoside	3.66	253, 354	479^−^	317	0.42 ^ab^ ± 0.15	0.24 ^a^ ± 0.09	0.56 ^b^ ± 0.06	0.21 ^a^ ± 0.09	0.38 ^a^ ± 0.17	0.60 ^b^ ± 0.10	0.43 ^b^ ± 0.11	0.55 ^b^ ± 0.09	1.03 ^c^ ± 0.06
18.	Quercetin 3-O-rutinoside	4.19	255, 354	609^−^	301	0.31 ^a^ ± 0.21	0.49 ^ab^ ± 0.14	0.42 ^a^ ± 0.02	0.73 ^b^ ± 0.13	0.66 ^b^ ± 0.03	0.49 ^ab^ ± 0.11	0.80 ^b^ ± 0.10	0.61 ^b^ ± 0.11	0.21 ^a^ ± 0.06
19.	Quercetin 3-O-glucoside	4.28	255, 355	463^−^	301	1.26 ^a^ ± 0.12	1.38 ^ab^ ± 0.21	1.70 ^bc^ ± 0.10	1.90 ^c^ ± 0.11	2.29 ^d^ ± 0.23	1.95 ^c^ ± 0.15	2.27 ^d^ ± 0.25	2.24 ^d^ ± 0.22	2.53 ^e^ ± 0.28
20.	Quercetin 3-O-galactoside	4.38	255, 355	463^−^	301	0.73 ^a^ ± 0.08	0.94 ^a^ ± 0.03	0.87 ^a^ ± 0.15	1.45 ^b^ ± 0.09	1.20 ^b^ ± 0.13	1.22 ^b^ ± 0.01	1.41 ^b^ ± 0.24	1.22 ^b^ ± 0.25	0.92 ^a^ ± 0.19
21.	Quercetin 3-O-pentoside I	4.63	255, 355	433^−^	301	0.11 ^a^ ± 0.01	0.17 ^a^ ± 0.00	0.16 ^a^ ± 0.08	0.24 ^a^ ± 0.02	0.23 ^a^ ± 0.09	0.22 ^a^ ± 0.05	0.26 ^a^ ± 0.09	0.22 ^a^ ± 0.06	0.04 ^a^ ± 0.00
22.	Quercetin 3-O-pentoside II	4.73	255, 355	433^−^	301	0.23 ^a^ ± 0.12	0.30 ^a^ ± 0.10	0.29 ^a^ ± 0.07	0.30 ^a^ ± 0.10	0.39 ^a^ ± 0.07	0.38 ^a^ ± 0.09	0.40 ^a^ ± 0.10	0.44 ^a^ ± 0.14	0.60 ^a^ ± 0.10
23.	Quercetin 3-O-pentoside III	4.76	255, 355	433^−^	301	0.28 ^a^ ± 0.07	0.36 ^b^ ± 0.11	0.21 ^a^ ± 0.11	0.19 ^a^ ± 0.06	0.28 ^a^ ± 0.01	0.45 ^b^ ± 0.13	0.51 ^b^ ± 0.11	0.52 ^b^ ± 0.12	0.35 ^ab^ ± 0.09
24.	Isorhamnetin 3-O-rutinoside	4.84	255, 355	623^−^	301	0.24 ^a^ ± 0.09	0.42 ^b^ ± 0.11	0.21 ^a^ ± 0.01	0.37 ^ab^ ± 0.03	0.41 ^b^ ± 0.11	0.10 ^a^ ± 0.00	0.48 ^b^ ± 0.15	0.33 ^a^ ± 0.01	0.16 ^a^ ± 0.04
25.	Quercetin 3-O-rhamnoside	4.95	255, 355	447^−^	301	0.47 ^ab^ ± 0.11	0.54 ^b^ ± 0.21	0.23 ^a^ ± 0.03	0.18 ^a^ ± 0.00	0.35 ^a^ ± 0.13	0.86 ^c^ ± 0.21	0.82 ^c^ ± 0.09	0.71 ^bc^ ± 0.16	0.57 ^b^ ± 0.14
26.	Quercetin 3-O-glucuronide	5.02	255, 354	477^−^	285	0.18 ^a^ ± 0.02	0.13 ^a^ ± 0.09	0.12 ^a^ ± 0.01	0.28 ^a^ ± 0.12	0.25 ^a^ ± 0.03	0.08 ^a^ ± 0.02	0.15 ^a^ ± 0.01	0.12 ^a^ ± 0.01	0.10 ^a^ ± 0.02
27.	Quercetin 3-O-(6′′-acetylo)-glucoside	5.08	255, 335	505^−^	301	0.43 ^b^ ± 0.06	0.54 ^b^ ± 0.12	0.30 ^ab^ ± 0.09	0.60 ^bc^ ± 0.14	0.59 ^b^ ± 0.11	0.60 ^bc^ ± 0.10	0.72 ^c^ ± 0.11	0.54 ^b^ ± 0.09	0.14 ^a^ ± 0.02
	Total					81.36 ^c^ ± 0.54	78.91 ^b^ ± 0.69	88.48 ^e^ ± 0.37	73.52 ^a^ ± 0.76	82.51 ^d^ ± 0.96	106.24 ^h^ ± 0.38	102.03 ^g^ ± 0.44	107.00 ^i^ ± 0.29	99.00 ^f^ ± 0.87

Data are expressed as mean values (*n* = 3) ± SD; SD—standard deviation. Mean values within rows with different letters are significantly different (*p* < 0.05) according to the Tukey test. + and − in MS column means ionisation mode—positive (+) and negative (−).

**Table A6:** Individual phenolic compounds identified by UPLC-PDA-MS/MS for clone BOR-21.

Compound		Rt	λ_max_	(M-H) m/z		Clone BOR-21								
(mg·100 g^−1^ d.w.)		(min)	(nm)	MS	MS/MS									
Storage Time (Days)						1			7			14		
Ozone Exposure Time (min)						0	15	30	0	15	30	0	15	30
Anthocyanins														
1.	Delphinidin-3-O-glucoside	2.22	276, 522	465^+^	303	14.80 ^g^ ± 0.26	5.81 ^b^ ± 0.11	8.52 ^d^ ± 0.22	3.79 ^a^ ± 0.09	7.36 ^c^ ± 0.14	15.24 ^h^ ± 0.12	9.04 ^e^ ± 0.21	9.22 ^e^ ± 0.25	14.22 ^f^ ± 0.31
2.	Delphinidin-3-O-galactoside	2.35	276, 522	465^+^	303	9.71 ^f^ ± 0.19	4.70 ^c^ ± 0.26	4.73 ^c^ ± 0.23	2.59 ^a^ ± 0.12	3.56 ^b^ ± 0.14	8.27 ^e^ ± 0.12	3.83 ^b^ ± 0.17	4.59 ^c^ ± 0.27	5.15 ^d^ ± 0.09
3.	Cyanidin-3-O-glucoside	2.61	276, 519	449^+^	287	12.18 ^f^ ± 0.15	5.83 ^b^ ± 0.21	6.80 ^c^ ± 0.13	3.93 ^a^ ± 0.16	6.61 ^c^ ± 0.21	12.56 ^g^ ± 0.06	7.72 ^d^ ± 0.15	7.46 ^d^ ± 0.25	11.74 ^e^ ± 0.25
4.	Cyanidin-3-O-galactoside	2.74	279, 516	449^+^	287	1.76 ^c^ ± 0.14	1.48 ^c^ ± 0.25	0.68 ^a^ ± 0.10	1.08 ^b^ ± 009	0.68 ^a^ ± 0.09	1.14 ^b^ ± 0.11	0.70 ^a^ ± 0.10	0.77 ^a^ ± 0.07	1.28 ^bc^ ± 0.13
5.	Petunidin-3-O-glucoside	2.80	276, 524	479^+^	317	8.29 ^f^ ± 0.17	3.72 ^b^ ± 0.11	5.02 ^c^ ± 0.22	3.28 ^a^ ± 0.09	5.32 ^cd^ ± 0.17	7.78 ^e^ ± 0.23	5.53 ^d^ ± 0.19	5.84 ^d^ ± 0.17	8.77 ^g^ ± 0.26
6.	Petunidin-3-O-galactoside	2.93	276, 522	479^+^	317	9.35 ^f^ ± 0.22	5.77 ^d^ ± 0.21	5.18 ^c^ ± 0.11	4.38 ^a^ ± 0.12	4.68 ^ab^ ± 0.21	7.79 ^e^ ± 0.19	4.71 ^b^ ± 0.19	5.55 ^d^ ± 0.25	5.06 ^c^ ± 0.21
7.	Petunidin-3-O-arabinoside	3.13	277, 526	449^+^	317	4.77 ^f^ ± 0.11	2.36 ^b^ ± 0.16	2.63 ^c^ ± 0.15	1.93 ^a^ ± 0.23	3.17 ^d^ ± 0.23	4.40 ^e^ ± 0.12	3.04 ^d^ ± 0.21	2.91 ^d^ ± 0.11	6.15 ^g^ ± 0.16
8.	Malvidin-3-O-glucoside	3.33	276, 524	493^+^	331	16.32 ^g^ ± 0.27	10.86 ^b^ ± 0.21	10.49 ^b^ ± 0.31	9.99 ^a^ ± 0.31	12.52 ^e^ ± 0.36	14.61 ^f^ ± 0.36	11.37 ^c^ ± 0.36	11.89 ^d^ ± 0.27	26.51 ^h^ ± 0.36
9.	Malvidin-3-O-galactoside	3.47	276, 526	493^+^	331	12.02 ^f^ ± 0.25	10.82 ^e^ ± 0.08	8.14 ^b^ ± 0.14	9.52 ^d^ ± 0.26	8.25 ^b^ ± 0.17	10.91 ^e^ ± 0.31	6.67 ^a^ ± 0.24	8.80 ^c^ ± 0.24	8.19 ^b^ ± 0.16
10.	Peonidin-3-O-glucoside	3.69	277, 527	463^+^	301	10.38 ^e^ ± 0.11	6.75 ^b^ ± 0.10	6.11 ^a^ ± 0.09	6.80 ^b^ ± 0.21	8.40 ^d^ ± 0.25	8.53 ^d^ ± 0.27	6.96 ^b^ ± 0.21	7.40 ^c^ ± 0.12	20.35 ^f^ ± 0.11
11.	Peonidin-3-O-galactoside	3.86	278, 527	463^+^	301	0.15 ^a^ ± 0.09	0.14 ^a^ ± 0.02	0.08 ^a^ ± 0.01	0.12 ^a^ ± 0.04	0.07 ^a^ ± 0.03	0.19 ^a^ ± 0.09	0.28 ^a^ ± 0.09	0.08 ^a^ ± 0.02	0.79 ^b^ ± 0.07
12.	Cyanidin-3-O-(6′′-acetyl)-glucoside	4.05	271, 520	491^+^	449, 287	1.24 ^c^ ± 0.23	1.04 ^c^ ± 0.13	0.43 ^a^ ± 0.09	0.60 ^b^ ± 0.11	0.29 ^a^ ± 0.15	0.33 ^a^ ± 0.11	0.38 ^a^ ± 0.11	0.27 ^a^ ± 0.05	0.44 ^ab^ ± 0.11
13.	Petunidin-3-O-(6′′-acetyl)-glucoside	4.19	277, 527	521^+^	479, 317	1.57 ^d^ ± 0.17	1.69 ^d^ ± 0.16	0.82 ^b^ ± 0.08	0.96 ^bc^ ± 0.15	0.33 ^a^ ± 0.09	1.18 ^c^ ± 0.11	0.87 ^b^ ± 0.13	0.41 ^a^ ± 0.10	1.67 ^d^ ± 0.15
14.	Cyanidin-3-O-(6′′-malonyl)glucoside	4.42	276, 529	535^+^	449, 287	0.68 ^ab^ ± 0.21	1.01 ^bc^ ± 0.09	0.56 ^a^ ± 0.10	0.91 ^b^ ± 0.13	0.51 ^a^ ± 0.14	0.88 ^b^ ± 0.15	1.21 ^c^ ± 0.11	0.59 ^a^ ± 0.11	7.40 ^d^ ± 0.16
15.	Cyanidin-3-O-(6′′-malonyl)galactoside	4.76	277, 529	535^+^	449, 287	2.42 ^c^ ± 0.23	4.42 ^e^ ± 0.15	1.69 ^b^ ± 0.15	2.93 ^d^ ± 0.13	1.02 ^a^ ± 0.11	2.42 ^c^ ± 0.21	1.65 ^b^ ± 0.22	1.20 ^a^ ± 0.10	6.62 ^f^ ± 0.24
Other Phenolics														
16.	Chlorogenic acid	2.68	288, 324	353^−^	191	16.27 ^d^ ± 0.34	17.14 ^e^ ± 0.27	15.68 ^c^ ± 0.29	18.63 ^f^ ± 0.22	15.40 ^bc^ ± 0.31	18.69 ^f^ ± 0.17	12.85 ^a^ ± 0.15	16.05 ^d^ ± 0.23	15.28 ^b^ ± 0.33
17.	Myricetin 3-O-glucoside	3.66	253, 354	479^−^	317	0.78 ^c^ ± 0.11	0.30 ^ab^ ± 0.09	0.74 ^c^ ± 0.04	0.11 ^a^ ± 0.02	0.33 ^b^ ± 0.11	1.10 ^d^ ± 0.11	0.34 ^b^ ± 0.12	0.36 ^b^ ± 0.09	0.70 ^c^ ± 0.10
18.	Quercetin 3-O-rutinoside	4.19	255, 354	609^−^	301	0.44 ^b^ ± 0.09	0.61 ^c^ ± 0.11	0.31 ^b^ ± 0.13	0.96 ^d^ ± 0.09	0.61 ^c^ ± 0.08	0.25 ^ab^ ± 0.05	0.51 ^bc^ ± 0.12	0.85 ^cd^ ± 0.14	0.08 ^a^ ± 0.02
19.	Quercetin 3-O-glucoside	4.28	255, 355	463^−^	301	2.40 ^c^ ± 0.18	1.93 ^b^ ± 0.13	1.71 ^b^ ± 0.15	3.31 ^d^ ± 0.21	1.97 ^b^ ± 0.22	2.58 ^c^ ± 0.24	0.91 ^a^ ± 0.13	2.42 ^c^ ± 0.21	1.16 ^a^ ± 0.11
20.	Quercetin 3-O-galactoside	4.38	255, 355	463^−^	301	1.29 ^bc^ ± 0.30	1.20 ^b^ ± 0.10	0.79 ^a^ ± 0.08	1.90 ^d^ ± 0.18	1.01 ^b^ ± 0.02	1.00 ^b^ ± 0.09	1.15 ^b^ ± 0.19	1.54 ^c^ ± 0.16	0.59 ^a^ ± 0.17
21.	Quercetin 3-O-pentoside I	4.63	255, 355	433^−^	301	0.19 ^a^ ± 0.08	0.19 ^a^ ± 0.07	0.07 ^a^ ± 0.00	0.39 ^b^ ± 0.06	0.20 ^a^ ± 0.07	0.12 ^a^ ± 0.02	0.10 ^a^ ± 0.02	0.22 ^ab^ ± 0.04	0.05 ^a^ ± 0.01
22.	Quercetin 3-O-pentoside II	4.73	255, 355	433^−^	301	0.40 ^b^ ± 0.10	0.33 ^b^ ± 0.03	0.37 ^b^ ± 0.09	0.53 ^b^ ± 0.14	0.34 ^b^ ± 0.12	0.48 ^b^ ± 0.21	0.13 ^a^ ± 0.05	0.41 ^b^ ± 0.11	0.36 ^b^ ± 0.09
23.	Quercetin 3-O-pentoside III	4.76	255, 355	433^−^	301	0.52 ^b^ ± 0.13	0.32 ^ab^ ± 0.11	0.26 ^a^ ± 0.06	0.16 ^a^ ± 0.01	0.27 ^a^ ± 0.08	0.30 ^a^ ± 0.03	0.17 ^a^ ± 0.01	0.42 ^b^ ± 0.08	0.33 ^ab^ ± 0.12
24.	Isorhamnetin 3-O-rutinoside	4.84	255, 355	623^−^	301	0.41 ^b^ ± 0.11	0.37 ^b^ ± 0.07	0.15 ^a^ ± 0.03	0.47 ^b^ ± 0.08	0.29 ^ab^ ± 0.11	0.19 ^a^ ± 0.03	0.21 ^a^ ± 0.07	0.31 ^ab^ ± 0.04	0.13 ^a^ ± 0.07
25.	Quercetin 3-O-rhamnoside	4.95	255, 355	447^−^	301	0.84 ^d^ ± 0.21	0.37 ^b^ ± 0.11	0.41 ^bc^ ± 0.12	0.08 ^a^ ± 0.02	0.38 ^b^ ± 0.10	0.58 ^c^ ± 0.14	0.16 ^a^ ± 0.05	0.59 ^cd^ ± 0.03	0.59 ^cd^ ± 0.13
26.	Quercetin 3-O-glucuronide	5.02	255, 354	477^−^	285	0.26 ^a^ ± 0.09	0.23 ^a^ ± 0.09	0.15 ^a^ ± 0.05	0.32 ^a^ ± 0.04	0.23 ^a^ ± 0.11	0.04 ^a^ ± 0.00	0.19 ^a^ ± 0.03	0.20 ^a^ ± 0.00	0.06 ^a^ ± 0.00
27.	Quercetin 3-O-(6′′-acetylo)-glucoside	5.08	255, 335	505^−^	301	0.68 ^c^ ± 0.09	0.43 ^bc^ ± 0.02	0.26 ^a^ ± 0.11	0.68 ^c^ ± 0.12	0.59 ^c^ ± 0.06	0.27 ^ab^ ± 0.02	0.36 ^b^ ± 0.10	0.68 ^c^ ± 0.14	0.06 ^a^ ± 0.02
	Total					130.12 ^g^ ± 0.93	89.82 ^d^ ± 0.42	82.78 ^b^ ± 0.66	80.35 ^a^ ± 0.36	84.39 ^c^ ± 0.71	121.82 ^f^ ± 0.88	81.05 ^a^ ± 0.31	91.04 ^e^ ± 0.58	143.73 ^h^ ± 0.68

Data are expressed as mean values (*n* = 3) ± SD; SD—standard deviation. Mean values within rows with different letters are significantly different (*p* < 0.05) according to the Tukey test. + and − in MS column means ionisation mode—positive (+) and negative (−).

**Table A7:** Individual phenolic compounds identified by UPLC-PDA-MS/MS for the standard cv. “Bluecrop”.

Compound		Rt	λ_max_	(M-H) m/z		Standard cv. “Bluecrop”								
(mg·100 g^−1^ d.w.)		(min)	(nm)	MS	MS/MS									
Storage Time (Days)						1			7			14		
Ozone Exposure Time (min)						0	15	30	0	15	30	0	15	30
Anthocyanins														
1.	Delphinidin-3-O-glucoside	2.22	276, 522	465^+^	303	7.44 ^b^ ± 0.15	7.49 ^b^ ± 0.22	7.42 ^b^ ± 0.13	9.10 ^d^ ± 0.17	8.07 ^c^ ± 0.05	16.66 ^f^ ± 0.32	6.18 ^a^ ± 0.13	7.11 ^b^ ± 0.11	11.66 ^e^ ± 0.14
2.	Delphinidin-3-O-galactoside	2.35	276, 522	465^+^	303	4.04 ^c^ ± 0.11	6.52 ^e^ ± 0.16	4.14 ^c^ ± 0.09	4.67 ^d^ ± 0.08	3.72 ^b^ ± 0.23	7.51 ^f^ ± 0.16	3.13 ^a^ ± 0.15	4.04 ^c^ ± 0.23	8.68 ^g^ ± 0.03
3.	Cyanidin-3-O-glucoside	2.61	276, 519	449^+^	287	6.69 ^c^ ± 0.27	7.65 ^d^ ± 0.15	6.33 ^b^ ± 0.23	7.25 ^d^ ± 0.24	6.16 ^b^ ± 0.14	12.96 ^f^ ± 0.21	5.77 ^a^ ± 0.09	7.34 ^d^ ± 0.18	10.75 ^e^ ± 0.31
4.	Cyanidin-3-O-galactoside	2.74	279, 516	449^+^	287	0.84 ^a^ ± 0.11	2.15 ^d^ ± 0.09	0.82 ^a^ ± 0.16	1.32 ^b^ ± 0.15	0.77 ^a^ ± 0.21	0.80 ^a^ ± 0.10	0.80 ^a^ ± 0.08	0.60 ^a^ ± 0.10	1.77 ^c^ ± 0.11
5.	Petunidin-3-O-glucoside	2.80	276, 524	479^+^	317	5.23 ^b^ ± 0.22	4.79 ^a^ ± 0.22	4.85 ^a^ ± 0.23	5.82 ^c^ ± 0.35	5.05 ^ab^ ± 0.03	9.02 ^e^ ± 0.17	4.77 ^a^ ± 0.25	4.88 ^a^ ± 0.15	6.19 ^d^ ± 0.14
6.	Petunidin-3-O-galactoside	2.93	276, 522	479^+^	317	5.48 ^c^ ± 0.18	7.68 ^f^ ± 0.28	4.93 ^b^ ± 0.27	5.96 ^d^ ± 0.27	4.33 ^a^ ± 0.15	7.22 ^e^ ± 0.21	4.84 ^b^ ± 0.27	5.06 ^b^ ± 0.06	8.71 ^g^ ± 0.13
7.	Petunidin-3-O-arabinoside	3.13	277, 526	449^+^	317	3.40 ^c^ ± 0.27	3.08 ^b^ ± 0.31	2.88 ^ab^ ± 0.15	2.58 ^a^ ± 0.24	2.53 ^a^ ± 0.07	4.91 ^e^ ± 0.17	2.71 ^a^ ± 0.21	3.34 ^c^ ± 0.13	4.17 ^d^ ± 0.09
8.	Malvidin-3-O-glucoside	3.33	276, 524	493^+^	331	12.28 ^b^ ± 0.17	14.43 ^d^ ± 0.25	10.75 ^a^ ± 0.19	12.23 ^b^ ± 0.13	10.53 ^a^ ± 0.41	15.14 ^e^ ± 0.11	10.86 ^a^ ± 0.16	15.30 ^e^ ± 0.20	13.30 ^c^ ± 0.10
9.	Malvidin-3-O-galactoside	3.47	276, 526	493^+^	331	9.40 ^c^ ± 0.11	13.29 ^f^ ± 0.33	8.12 ^b^ ± 0.12	9.41 ^c^ ± 0.16	7.37 ^a^ ± 0.15	9.59 ^cd^ ± 0.16	8.30 ^b^ ± 0.10	9.96 ^d^ ± 0.13	12.46 ^e^ ± 0.01
10.	Peonidin-3-O-glucoside	3.69	277, 527	463^+^	301	8.55 ^d^ ± 0.16	9.11 ^e^ ± 0.21	7.32 ^c^ ± 0.22	6.09 ^b^ ± 0.09	5.78 ^a^ ± 0.26	9.71 ^f^ ± 0.23	7.67 ^c^ ± 0.17	13.55 ^h^ ± 0.25	10.29 ^g^ ± 0.06
11.	Peonidin-3-O-galactoside	3.86	278, 527	463^+^	301	0.15 ^a^ ± 0.21	0.23 ^a^ ± 0.07	0.25 ^a^ ± 0.09	0.23 ^a^ ± 0.07	0.17 ^a^ ± 0.04	0.08 ^a^ ± 0.00	0.20 ^a^ ± 0.07	0.19 ^a^ ± 0.09	0.20 ^a^ ± 0.07
12.	Cyanidin-3-O-(6′′-acetyl)-glucoside	4.05	271, 520	491^+^	449, 287	0.86 ^b^ ± 0.14	1.21 ^c^ ± 0.11	0.76 ^b^ ± 0.07	0.74 ^b^ ± 0.03	0.48 ^a^ ± 0.21	0.35 ^a^ ± 0.08	0.56 ^ab^ ± 0.11	0.49 ^a^ ± 0.12	1.54 ^d^ ± 0.15
13.	Petunidin-3-O-(6′′-acetyl)-glucoside	4.19	277, 527	521^+^	479, 317	0.92 ^b^ ± 0.10	1.96 ^d^ ± 0.16	1.63 ^c^ ± 0.33	1.08 ^b^ ± 0.12	0.81 ^b^ ± 0.15	0.46 ^a^ ± 0.11	0.94 ^b^ ± 0.10	1.07 ^b^ ± 0.08	1.94 ^d^ ± 0.11
14.	Cyanidin-3-O-(6′′-malonyl)glucoside	4.42	276, 529	535^+^	449, 287	0.89 ^b^ ± 0.11	1.45 ^d^ ± 0.13	1.22 ^cd^ ± 0.15	1.17 ^c^ ± 0.12	0.89 ^b^ ± 0.06	0.51 ^a^ ± 0.08	1.14 ^c^ ± 0.15	1.42 ^d^ ± 0.14	1.12 ^c^ ± 0.09
15.	Cyanidin-3-O-(6′′-malonyl)galactoside	4.76	277, 529	535^+^	449, 287	1.75 ^b^ ± 0.11	5.05 ^f^ ± 0.06	3.51 ^e^ ± 0.14	2.76 ^d^ ± 0.31	2.18 ^c^ ± 0.11	1.05 ^a^ ± 0.11	1.98 ^bc^ ± 0.18	2.54 ^d^ ± 0.16	3.88 ^e^ ± 0.22
Other Phenolics														
16.	Chlorogenic acid	2.68	288, 324	353^−^	191	18.79 ^e^ ± 0.24	16.03 ^a^ ± 0.25	16.10 ^a^ ± 0.28	16.70 ^bc^ ± 0.20	16.67 ^b^ ± 0.31	16.93 ^c^ ± 0.12	17.87 ^d^ ± 0.16	16.67 ^b^ ± 0.07	16.15 ^a^ ± 0.34
17.	Myricetin 3-O-glucoside	3.66	253, 354	479^−^	317	0.24 ^a^ ± 0.05	0.50 ^b^ ± 0.12	0.61 ^b^ ± 0.17	0.30 ^a^ ± 0.08	0.27 ^a^ ± 0.06	0.61 ^b^ ± 0.09	0.35 ^ab^ ± 0.07	0.67 ^b^ ± 0.18	0.75 ^b^ ± 0.13
18.	Quercetin 3-O-rutinoside	4.19	255, 354	609^−^	301	0.78 ^b^ ± 0.08	0.60 ^b^ ± 0.10	0.64 ^b^ ± 0.18	0.80 ^b^ ± 0.16	0.63 ^b^ ± 0.12	0.23 ^a^ ± 0.04	0.63 ^b^ ± 0.08	0.28 ^a^ ± 0.07	0.63 ^b^ ± 0.11
19.	Quercetin 3-O-glucoside	4.28	255, 355	463^−^	301	2.55 ^b^ ± 0.21	1.63 ^a^ ± 0.08	2.28 ^b^ ± 0.08	2.43 ^b^ ± 0.14	2.28 ^b^ ± 0.12	1.39 ^a^ ± 0.11	2.35 ^b^ ± 0.03	1.34 ^a^ ± 0.11	2.69 ^b^ ± 0.13
20.	Quercetin 3-O-galactoside	4.38	255, 355	463^−^	301	1.73 ^c^ ± 0.13	1.29 ^b^ ± 0.13	1.39 ^bc^ ± 0.12	1.61 ^c^ ± 0.18	1.31 ^b^ ± 0.01	0.85 ^a^ ± 0.17	1.57 ^c^ ± 0.14	0.92 ^a^ ± 0.19	1.63 ^c^ ± 0.11
21.	Quercetin 3-O-pentoside I	4.63	255, 355	433^−^	301	0.33 ^a^ ± 0.05	0.12 ^a^ ± 0.01	0.17 ^a^ ± 0.09	0.29 ^a^ ± 0.10	0.25 ^a^ ± 0.15	0.10 ^a^ ± 0.01	0.21 ^a^ ± 0.10	0.11 ^a^ ± 0.01	0.21 ^a^ ± 0.07
22.	Quercetin 3-O-pentoside II	4.73	255, 355	433^−^	301	0.39 ^a^ ± 0.09	0.26 ^a^ ± 0.04	0.42 ^a^ ± 0.14	0.42 ^a^ ± 0.13	0.38 ^a^ ± 0.09	0.25 ^a^ ± 0.08	0.37 ^a^ ± 0.11	0.24 ^a^ ± 0.04	0.53 ^a^ ± 0.13
23.	Quercetin 3-O-pentoside III	4.76	255, 355	433^−^	301	0.12 ^a^ ± 0.01	0.15 ^a^ ± 0.02	0.26 ^a^ ± 0.09	0.31 ^ab^ ± 0.06	0.22 ^a^ ± 0.02	0.27 ^a^ ± 0.03	0.22 ^a^ ± 0.06	0.28 ^a^ ± 0.09	0.54 ^b^ ± 0.09
24.	Isorhamnetin 3-O-rutinoside	4.84	255, 355	623^−^	301	0.40 ^a^ ± 0.10	0.26 ^a^ ± 0.05	0.13 ^a^ ± 0.02	0.48 ^a^ ± 0.09	0.35 ^a^ ± 0.07	0.17 ^a^ ± 0.00	0.26 ^a^ ± 0.06	0.14 ^a^ ± 0.02	0.10 ^a^ ± 0.08
25.	Quercetin 3-O-rhamnoside	4.95	255, 355	447^−^	301	0.04 ^a^ ± 0.00	0.18 ^a^ ± 0.08	0.58 ^b^ ± 0.11	0.30 ^b^ ± 0.10	0.28 ^ab^ ± 0.03	0.46 ^b^ ± 0.08	0.29 ^b^ ± 0.09	0.53 ^b^ ± 0.13	0.53 ^b^ ± 0.10
26.	Quercetin 3-O-glucuronide	5.02	255, 354	477^−^	285	0.36 ^a^ ± 0.03	0.26 ^a^ ± 0.09	0.14 ^a^ ± 0.07	0.33 ^a^ ± 0.03	0.25 ^a^ ± 0.05	0.12 ^a^ ± 0.02	0.24 ^a^ ± 0.00	0.17 ^a^ ± 0.05	0.23 ^a^ ± 0.00
27.	Quercetin 3-O-(6′′-acetylo)-glucoside	5.08	255, 335	505^−^	301	0.62 ^b^ ± 0.15	0.48 ^b^ ± 0.15	0.36 ^ab^ ± 0.10	0.74 ^b^ ± 0.09	0.56 ^b^ ± 0.16	0.26 ^a^ ± 0.02	0.52 ^b^ ± 0.13	0.31 ^a^ ± 0.09	0.49 ^b^ ± 0.05
	Total					94.25 ^d^ ± 0.77	107.88 ^f^ ± 0.51	88.02 ^c^ ± 0.33	95.12 ^d^ ± 0.82	82.31 ^a^ ± 0.55	117.58 ^g^ ± 0.68	84.75 ^b^ ± 0.45	98.54 ^e^ ± 0.72	121.16 ^h^ ± 0.27

Data are expressed as mean values (*n* = 3) ± SD; SD—standard deviation. Mean values within rows with different letters are significantly different (*p* < 0.05) according to the Tukey test. + and − in MS column means ionisation mode—positive (+) and negative (−).

**Table A8:** Individual phenolic compounds identified by UPLC-PDA-MS/MS for the standard cv. “Duke”.

Compound		Rt	λ_max_	(M-H) m/z		Standard cv. “Duke”								
(mg·100 g^−1^ d.w.)		(min)	(nm)	MS	MS/MS									
Storage Time (Days)						1			7			14		
Ozone Exposure Time (min)						0	15	30	0	15	30	0	15	30
Anthocyanins														
1.	Delphinidin-3-O-glucoside	2.22	276, 522	465^+^	303	11.30 ^d^ ± 0.15	8.94 ^c^ ± 0.11	6.62 ^b^ ± 0.07	5.74 ^a^ ± 0.14	6.30 ^b^ ± 0.10	17.84 ^h^ ± 0.22	14.22 ^g^ ± 0.12	12.12 ^e^ ± 0.11	13.12 ^f^ ± 0.02
2.	Delphinidin-3-O-galactoside	2.35	276, 522	465^+^	303	5.46 ^e^ ± 0.09	4.14 ^c^ ± 0.08	3.74 ^b^ ± 0.05	3.25 ^a^ ± 0.10	3.69 ^b^ ± 0.15	7.36 ^g^ ± 0.18	6.26 ^f^ ± 0.07	4.45 ^d^ ± 0.13	6.63 ^f^ ± 0.11
3.	Cyanidin-3-O-glucoside	2.61	276, 519	449^+^	287	7.92 ^c^ ± 0.10	7.52 ^c^ ± 0.17	5.71 ^b^ ± 0.10	5.29 ^a^ ± 0.06	5.68 ^b^ ± 0.09	12.60 ^e^ ± 0.13	11.12 ^d^ ± 0.14	11.03 ^d^ ± 0.05	11.32 ^d^ ± 0.08
4.	Cyanidin-3-O-galactoside	2.74	279, 516	449^+^	287	0.70 ^a^ ± 0.02	0.93 ^ab^ ± 0.01	0.94 ^ab^ ± 0.09	0.75 ^a^ ± 0.07	0.86 ^a^ ± 0.00	0.62 ^a^ ± 0.02	1.28 ^b^ ± 0.09	2.21 ^c^ ± 0.11	1.24 ^b^ ± 0.14
5.	Petunidin-3-O-glucoside	2.80	276, 524	479^+^	317	6.14 ^c^ ± 017	5.61 ^b^ ± 0.01	5.28 ^b^ ± 0.08	4.28 ^a^ ± 0.05	4.22 ^a^ ± 0.12	8.58 ^e^ ± 0.11	7.82 ^d^ ± 0.12	6.46 ^c^ ± 0.15	7.55 ^d^ ± 0.09
6.	Petunidin-3-O-galactoside	2.93	276, 522	479^+^	317	5.65 ^b^ ± 0.12	5.34 ^b^ ± 0.06	5.55 ^b^ ± 0.20	4.51 ^a^ ± 0.11	4.66 ^a^ ± 0.06	6.76 ^c^ ± 0.16	7.07 ^d^ ± 0.09	4.44 ^a^ ± 0.09	6.92 ^c^ ± 0.03
7.	Petunidin-3-O-arabinoside	3.13	277, 526	449^+^	317	3.07 ^c^ ± 0.09	2.90 ^bc^ ± 0.12	2.71 ^b^ ± 0.12	2.52 ^ab^ ± 0.18	2.24 ^a^ ± 0.23	4.32 ^e^ ± 0.13	3.74 ^d^ ± 0.16	5.81 ^g^ ± 0.22	5.04 ^f^ ± 0.14
8.	Malvidin-3-O-glucoside	3.33	276, 524	493^+^	331	11.68 ^d^ ± 0.11	10.58 ^b^ ± 0.15	12.73 ^f^ ± 0.13	11.03 ^c^ ± 0.03	8.80 ^a^ ± 0.21	12.38 ^e^ ± 0.09	13.41 ^g^ ± 0.02	21.15 ^i^ ± 0.17	13.88 ^h^ ± 0.21
9.	Malvidin-3-O-galactoside	3.47	276, 526	493^+^	331	8.24 ^d^ ± 0.14	7.32 ^b^ ± 0.19	10.00 ^f^ ± 0.11	8.14 ^cd^ ± 0.02	6.88 ^a^ ± 0.17	7.96 ^c^ ± 0.11	7.96 ^c^ ± 0.08	6.84 ^a^ ± 0.11	9.60 ^e^ ± 0.05
10.	Peonidin-3-O-glucoside	3.69	277, 527	463^+^	301	6.13 ^b^ ± 0.08	6.44 ^bc^ ± 0.22	7.62 ^e^ ± 0.22	8.00 ^f^ ± 0.14	5.30 ^a^ ± 0.14	6.85 ^c^ ± 0.15	7.16 ^d^ ± 0.10	17.87 ^h^ ± 0.12	9.43 ^g^ ± 0.01
11.	Peonidin-3-O-galactoside	3.86	278, 527	463^+^	301	0.08 ^a^ ± 0.01	0.19 ^a^ ± 0.09	0.09 ^a^ ± 0.00	0.12 ^a^ ± 0.03	0.14 ^a^ ± 0.03	0.06 ^a^ ± 0.00	0.44 ^b^ ± 0.08	0.67 ^b^ ± 0.04	0.07 ^a^ ± 0.01
12.	Cyanidin-3-O-(6′′-acetyl)-glucoside	4.05	271, 520	491^+^	449, 287	0.59 ^a^ ± 0.09	0.31 ^a^ ± 0.01	0.43 ^a^ ± 0.03	0.28 ^a^ ± 0.11	0.43 ^a^ ± 0.03	0.51 ^a^ ± 0.17	0.68 ^a^ ± 0.06	0.26 ^a^ ± 0.01	0.44 ^a^ ± 0.04
13.	Petunidin-3-O-(6′′-acetyl)-glucoside	4.19	277, 527	521^+^	479, 317	0.61 ^a^ ± 0.00	0.69 ^a^ ± 0.11	0.90 ^b^ ± 0.15	0.86 ^b^ ± 0.07	0.82 ^ab^ ± 0.08	0.50 ^a^ ± 0.10	1.29 ^c^ ± 0.09	1.36 ^c^ ± 0.12	0.47 ^a^ ± 0.07
14.	Cyanidin-3-O-(6′′-malonyl)glucoside	4.42	276, 529	535^+^	449, 287	0.37 ^a^ ± 0.07	0.85 ^b^ ± 0.05	0.67 ^ab^ ± 0.17	1.01 ^c^ ± 0.10	0.72 ^b^ ± 0.12	0.22 ^a^ ± 0.02	1.52 ^d^ ± 0.11	5.34 ^e^ ± 0.11	0.38 ^a^ ± 0.05
15.	Cyanidin-3-O-(6′′-malonyl)galactoside	4.76	277, 529	535^+^	449, 287	1.03 ^bc^ ± 0.11	1.26 ^c^ ± 0.16	1.59 ^d^ ± 0.09	1.87 ^d^ ± 0.11	1.41 ^cd^ ±	0.53 ^a^ ± 0.12	2.32 ^e^ ± 0.06	5.00 ^f^ ± 0.23	0.99 ^b^ ± 0.22
Other Phenolics														
16.	Chlorogenic acid	2.68	288, 324	353^−^	191	16.00 ^b^ ± 0.34	14.39 ^b^ ± 0.23	13.09 ^a^ ± 0.22	15.28 ^c^ ± 0.18	15.59 ^cd^ ± 0.16	15.99 ^d^ ± 0.22	18.42 ^g^ ± 0.09	16.69 ^f^ ± 0.21	16.74 ^f^ ± 0.22
17.	Myricetin 3-O-glucoside	3.66	253, 354	479^−^	317	0.43 ^a^ ± 0.21	0.42 ^ab^ ± 0.14	0.22 ^a^ ± 0.02	0.64 ^b^ ± 0.03	0.29 ^a^ ± 0.13	0.29 ^a^ ± 0.03	0.84 ^b^ ± 0.09	0.18 ^a^ ± 0.02	0.58 ^b^ ± 0.08
18.	Quercetin 3-O-rutinoside	4.19	255, 354	609^−^	301	0.71 ^ab^ ± 0.18	0.42 ^a^ ± 0.15	0.49 ^a^ ± 0.07	0.66 ^a^ ± 0.06	0.72 ^a^ ± 0.15	0.74 ^a^ ± 0.11	0.65 ^a^ ± 0.03	0.73 ^a^ ± 0.09	0.52 ^a^ ± 0.11
19.	Quercetin 3-O-glucoside	4.28	255, 355	463^−^	301	1.49 ^a^ ± 0.24	0.98 ^a^ ± 0.22	1.22 ^a^ ± 0.08	2.18 ^cd^ ± 0.13	1.61 ^b^ ± 0.11	2.10 ^c^ ± 0.10	2.42 ^d^ ± 0.16	1.91 ^c^ ± 0.11	1.75 ^bc^ ± 0.05
20.	Quercetin 3-O-galactoside	4.38	255, 355	463^−^	301	0.97 ^ab^ ± 0.08	0.66 ^a^ ± 0.13	1.04 ^b^ ± 0.04	1.44 ^c^ ± 0.12	1.29 ^bc^ ± 0.11	1.14 ^b^ ± 0.15	1.48 ^c^ ± 0.08	1.44 ^c^ ± 0.08	1.07 ^b^ ± 0.11
21.	Quercetin 3-O-pentoside I	4.63	255, 355	433^−^	301	0.21 ^a^ ± 0.09	0.14 ^a^ ± 0.05	0.12 ^a^ ± 0.02	0.16 ^a^ ± 0.04	0.12 ^a^ ± 0.01	0.24 ^a^ ± 0.00	0.23 ^a^ ± 0.13	0.21 ^a^ ± 0.01	0.19 ^a^ ± 0.03
22.	Quercetin 3-O-pentoside II	4.73	255, 355	433^−^	301	0.28 ^a^ ± 0.01	0.19 ^a^ ± 0.10	0.21 ^a^ ± 0.11	0.40 ^a^ ± 0.08	0.26 ^a^ ± 0.06	0.28 ^a^ ± 0.08	0.42 ^a^ ± 0.14	0.36 ^a^ ± 0.06	0.33 ^a^ ± 0.12
23.	Quercetin 3-O-pentoside III	4.76	255, 355	433^−^	301	0.34 ^a^ ± 0.16	0.34 ^b^ ± 0.11	0.17 ^a^ ± 0.07	0.12 ^a^ ± 0.02	0.38 ^b^ ± 0.12	0.30 ^ab^ ± 0.10	0.30 ^ab^ ± 0.07	0.30 ^ab^ ± 0.05	0.42 ^b^ ± 0.09
24.	Isorhamnetin 3-O-rutinoside	4.84	255, 355	623^−^	301	0.37 ^b^ ± 0.21	0.32 ^a^ ± 0.02	0.18 ^a^ ± 0.11	0.15 ^a^ ± 0.07	0.29 ^a^ ± 0.10	0.35 ^a^ ± 0.00	0.14 ^a^ ± 0.02	0.38 ^a^ ± 0.11	0.24 ^a^ ± 0.06
25.	Quercetin 3-O-rhamnoside	4.95	255, 355	447^−^	301	0.41 ^b^ ± 0.09	0.49 ^b^ ± 0.09	0.18 ^a^ ± 0.06	0.34 ^a^ ± 0.04	0.58 ^b^ ± 0.14	0.52 ^b^ ± 0.12	0.44 ^ab^ ± 0.17	0.30 ^a^ ± 0.09	0.62 ^b^ ± 0.13
26.	Quercetin 3-O-glucuronide	5.02	255, 354	477^−^	285	0.24 ^a^ ± 0.07	0.17 ^a^ ± 0.18	0.17 ^a^ ± 0.10	0.15 ^a^ ± 0.15	0.20 ^a^ ± 0.11	0.28 ^a^ ± 0.14	0.05 ^a^ ± 0.01	0.26 ^a^ ± 0.06	0.18 ^a^ ± 0.10
27.	Quercetin 3-O-(6′′-acetylo)-glucoside	5.08	255, 335	505^−^	301	0.61 ^b^ ± 0.01	0.45 ^ab^ ± 0.05	0.41 ^a^ ± 0.06	0.24 ^a^ ± 0.10	0.58 ^b^ ± 0.08	0.72 ^b^ ± 0.19	0.19 ^a^ ± 0.08	0.65 ^b^ ± 0.12	0.55 ^b^ ± 0.05
	Total					101.97 ^g^ ± 0.49	87.95 ^d^ ± 0.88	80.51 ^b^ ± 0.32	86.33 ^c^ ± 0.66	79.55 ^b^ ± 0.41	75.11 ^a^ ± 0.82	112.68 ^f^ ± 0.95	109.71 ^e^ ± 0.77	128.21 ^g^ ± 0.69

Data are expressed as mean values (*n* = 3) ± SD; SD—standard deviation. Mean values within rows with different letters are significantly different (*p* < 0.05) according to the Tukey test. + and − in MS column means ionisation mode—positive (+) and negative (−).
